# Neuromorphic Computing-Assisted Triboelectric Capacitive-Coupled Tactile Sensor Array for Wireless Mixed Reality Interaction

**DOI:** 10.1021/acsnano.4c03554

**Published:** 2024-06-21

**Authors:** Xinkai Xie, Qinan Wang, Chun Zhao, Qilei Sun, Haicheng Gu, Junyan Li, Xin Tu, Baoqing Nie, Xuhui Sun, Yina Liu, Eng Gee Lim, Zhen Wen, Zhong Lin Wang

**Affiliations:** † Institute of Functional Nano and Soft Materials (FUNSOM), Joint International Research Laboratory of Carbon-Based Functional Materials and Devices, Soochow University, Suzhou 215123, P. R. China; ‡ Department of Electrical and Electronic Engineering, School of Advanced Technology, Xi’an Jiaotong-Liverpool University, Suzhou 215123, P. R. China; § Department of Applied Mathematics, School of Mathematics and Physics, Xi’an Jiaotong-Liverpool University, Suzhou 215123, P. R. China; ∥ Department of Electrical and Electronic Engineering, University of Liverpool, Liverpool L693GJ, U.K.; ⊥ School of Electronic and Information Engineering, Soochow University, Suzhou 215006, P. R. China; # Beijing Institute of Nanoenergy and Nanosystems, Chinese Academy of Sciences, Beijing 101400, P. R. China; ¶ School of Materials Science and Engineering, Georgia Institute of Technology, Atlanta, Georgia 30332-0245, United States; ∇ Joint International Research Laboratory of Information Display and Visualization, School of Electronic Science and Engineering, Southeast University, Nanjing 210096, P. R. China

**Keywords:** triboelectric-capacitive-coupled, tactile sensor array, neuromorphic computation, human–machine interface, mixed reality

## Abstract

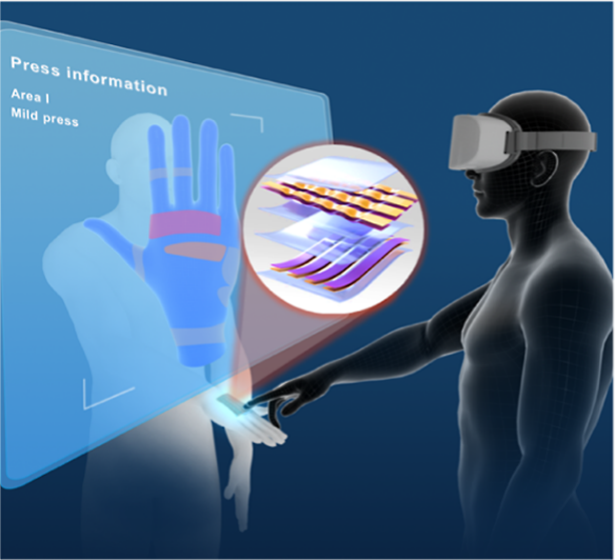

Flexible tactile sensors show promise for artificial intelligence applications due to their biological adaptability and rapid signal perception. Triboelectric sensors enable active dynamic tactile sensing, while integrating static pressure sensing and real-time multichannel signal transmission is key for further development. Here, we propose an integrated structure combining a capacitive sensor for static spatiotemporal mapping and a triboelectric sensor for dynamic tactile recognition. A liquid metal-based flexible dual-mode triboelectric-capacitive-coupled tactile sensor (TCTS) array of 4 × 4 pixels achieves a spatial resolution of 7 mm, exhibiting a pressure detection limit of 0.8 Pa and a fast response of 6 ms. Furthermore, neuromorphic computing using the MXene-based synaptic transistor achieves 100% recognition accuracy of handwritten numbers/letters within 90 epochs based on dynamic triboelectric signals collected by the TCTS array, and cross-spatial information communication from the perceived multichannel tactile data is realized in the mixed reality space. The results illuminate considerable application possibilities of dual-mode tactile sensing technology in human–machine interfaces and advanced robotics.

## Introduction

A tactile sensor enabled diverse applications in soft robotics, human–machine interface, environmental monitoring, health care, etc.^1−3^ Triboelectric sensors originating from the Maxwell displacement current could actively convert mechanical energy into electricity, providing an effective approach to self-powered dynamic tactile sensing, which features high sensitivity, broad material availability, and ease-of-manufacturing.^4−6^ However, how to obtain stable static pressure detection on the same device is a key scientific issue for the application of triboelectric tactile sensors in the field of artificial intelligence of things (AIoT).^7−9^ Conventional passive capacitive sensors could be utilized to detect static pressures. Based on the intrinsic capacitor model of TENG, integration of a triboelectric sensor with the traditional passive capacitive sensor could become an effective strategy to perceive both static and dynamic tactile signals, presenting advantages of accurate real-time pressure monitoring, low power consumption, and simple circuit signal processing.^10,11^

Based on tactile sensing systems and the machine learning method, virtual reality (VR) technology brings users an immersive experience by establishing a three-dimensional environment and creating a technique for human–machine interfaces (HMI).^12,13^ Human judgment on objects depending on multiple sensory information from different modalities can improve estimation accuracy.^14−16^ Specific to the combined effect of vision and touch, although visual information can be obtained directly, obtaining real-time tactile feedback is more important for remote operation and training in virtual environments. For instance, F. Wen et al. proposed a sign language recognition and communication system comprising a smart triboelectric glove, AI block, and the back-end VR interface.^17^ Furthermore, M. Zhu et al. designed a triboelectric bidirectional sensor that can be universally applied on different joints of the exoskeleton arm for capturing and projecting the motions of the entire upper limbs and playing table tennis games.^18^ However, several challenges occur, such as access to both virtual and real models, multichannel information perception, and quantitative visualization of the output.

Different from the completely virtual world of VR, mixed reality (MR) technology presents “real” and “virtual” interactivity. MR refers to an immersive technology that combines elements of both VR and augmented reality (AR). It blends digital content with the real-world environment that allows users to manipulate virtual objects while still being aware of and able to interact with the physical world. This reality technology is enabled by advancements in computer vision, graphics processing, input systems, and cloud computing that offers environmental input and perception. Furthermore, in practical applications of tactile sensors in the field of AIoT, the processing flow of output signals is complicated, time-consuming, and inefficient.^19^ The combination with the optimized machine learning model based on the neuromorphic computing method could shorten the signal processing time and improve the recognition accuracy, which is expected to be deeply integrated with HMI.^20−22^ Synaptic transistors based on 2D materials feature short ion transport distance, excellent electron transport dynamics, and high mobility, playing as an important role of neuromorphic computing.^23−25^ The working mechanism is coupling of ion migration and electron–hole pairs generation.^26,27^

In this work, we propose a dual-mode flexible triboelectric-capacitive-coupled tactile sensor (TCTS) array enabled by cross-stacked EGaIn coated stripe electrodes and silicone rubber encapsulation. The TCTS is composed of 4 × 4 sensing units with a total of 16 pixels and a spatial resolution of 7 mm for tactile pressure mapping and recognition. For the capacitive sensing mode, the capacitance variation of the TCTS unit is modulated in the range from 5.4 to 19.1 pF (0–80 kPa). By placing different weights on the sensor array, we can realize a contour pattern visualization of the pressure-capacitance mapping. For the triboelectric sensing mode, the TCTS unit apprehends the output voltage response sensitivity of 7.88 kPa^–1^ in the small pressure range (0–8.78 kPa). The detection limit is as low as 0.8 Pa with a fast response of 6 ms. As a favorable application for AI-enabled tactile sensing, the synaptic transistor-based neuromorphic-computing method for artificial neural network (ANN) recognition of complex handwritten input signals is achieved with a high accuracy of 100%. Toward the feasible application of MR, the TCTS array can be functionalized as the multichannel tactile sensing for acupressure intensities, rendering visual instruction of light-to-dark color transitions. Hence, a comprehensive, realistic, and immersive three-dimensional mixed-reality sensory interaction system is constructed, which can benefit telemedicine, equipment manufacturing, education, entertainment, etc.

## Results and Discussion

### Design of TCTS Array Enabled MR Interaction and Sensory-Neuromorphic System

**Figure 1:**
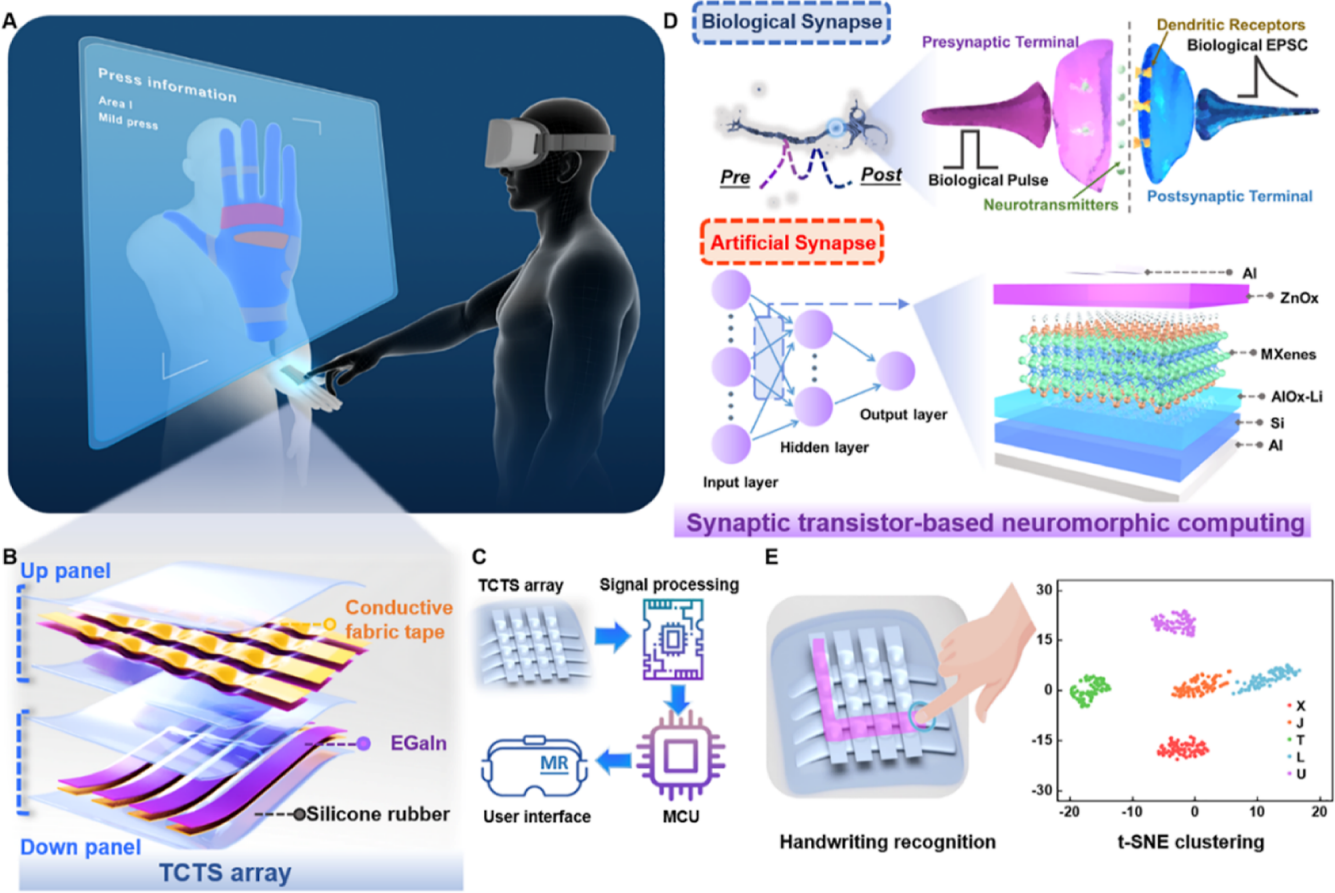
Schematic diagram of the machine-learning-assisted triboelectric-capacitive coupled tactile sensor (TCTS) array enabled MR interaction. (A) Configuration of the system showing press strength perception feedback on the hand with the TCTS array in the AR space. (B) Structure of the TCTS array, including the top panel of silicone rubber encapsulated arch-shaped EgaIn electrodes with parallel array pattern coated on conductive fabric tape and the bottom panel of encapsulated flat electrodes, overlapping to form 16 unit pixels. (C) Overview of the TCTS enabled acupressure application in MR interfaces. (D) Schematic illustration of synaptic transistor based neuromorphic computing strategy. (E) t-SNE clustering results of handwriting recognition.

MR is expected to realize AIoT applications by constructing real-object models on the virtual display and the projection of objects in real scenes through model tracking technology. Figure 1A depicts the system showing press strength perception feedback on different areas of the hand with a triboelectric-capacitive-coupled tactile sensor (TCTS) array in the MR space. The virtual model of a real hand and the different areas on the back of the hand in the real environment are created. As the user wears the MR device, both the real environment and the constructed virtual model can be observed on the display. The virtual model can be tracked to the real human hand and perfectly overlapped with it. Here, the TCTS is placed on the real hand, which covers different areas. When the user presses on one area of the hand, multichannel information about the area and pressure intensity could be observed, for example, Area I, mild press. The structural design and optical photograph of the TCTS array are shown in Figures 1B and S1, which is composed of the two panels crossly stacked to form a 4 × 4 matrix of 16 pixels. The fabrication process flow of the TCTS array is depicted in Figure S2. Four stripe arch-shaped electrodes coated with EGaIn are wrapped by silicone rubber to form the up panel. Considering the stability of the precise cross-alignment and mutual contact of the upper and lower strip electrodes, the bottom panel adopts flat strip electrodes. EGaIn features high conductivity and low Young’s modulus, and the oxide layer formed on its surface serves as an intermediate layer to further enhance the output, making it a robust candidate for the flexible TCTS electrode. EGaIn is sprayed to evenly distribute it on the surface of flexible conductive tape, which greatly reduces the thickness of the electrode layer and improves the safety. In addition, the top striped arch electrode and the hemispherical dielectric structure design show stretchability and increase the sensitivity of the sensor unit. To take advantage of the multichannel tactile sensing property of our flexible artificial skin, we utilized the TCTS array to create an MR interactive human–machine interface for perceiving distinct touching areas and different pressure levels. As a concept flow shown in Figure 1C, tactile information data are initially captured by the TCTS array and then cascade-amplified through the signal processing circuit, converted into a digital signal via a microcontroller unit, and transmitted to the Unity platform through bluetooth for displaying the tactile message on the MR device terminal. Inspired by the biological synapses shown in Figure 1D, artificial synapses mimic their functions of signal transmission between neurons for synaptic plasticity and learning behavior, which serve as core components of neuromorphic computing. Since the processing flow of the output signals from the tactile sensor array is complicated, time-consuming, and inefficient, a combination of perceived tactile information and the optimized neuromorphic computing strategy could shorten the signal processing time and improve the recognition accuracy. Therefore, we establish an ANN based on the three terminal synaptic transistor with the all-in-one structure of Al/ZnO_*x*_/MXenes/AlO_*x*_-Li/Si/Al. Two-dimensional material, for example, MXene has the advantages of short ion transport distance, excellent electron transport dynamics, and high mobility to form a synaptic device, demonstrating excellent synaptic plasticity and learning capabilities. The synaptic weights between artificial neurons are represented by the conductance values of the synaptic transistor for ANN training. For demonstration, the TCTS array could be utilized as a flexible handwriting panel, where the collected chronological signal data set is accurately categorized into manual letters through an ANN enabled by the proposed synaptic transistor and then realizing visualization by t-distributed Stochastic Neighbor Embedding (t-SNE) dimensional degradation strategy (Figure 1E).

### Working Mechanism of Dual-Mode TCTS Array

**Figure 2:**
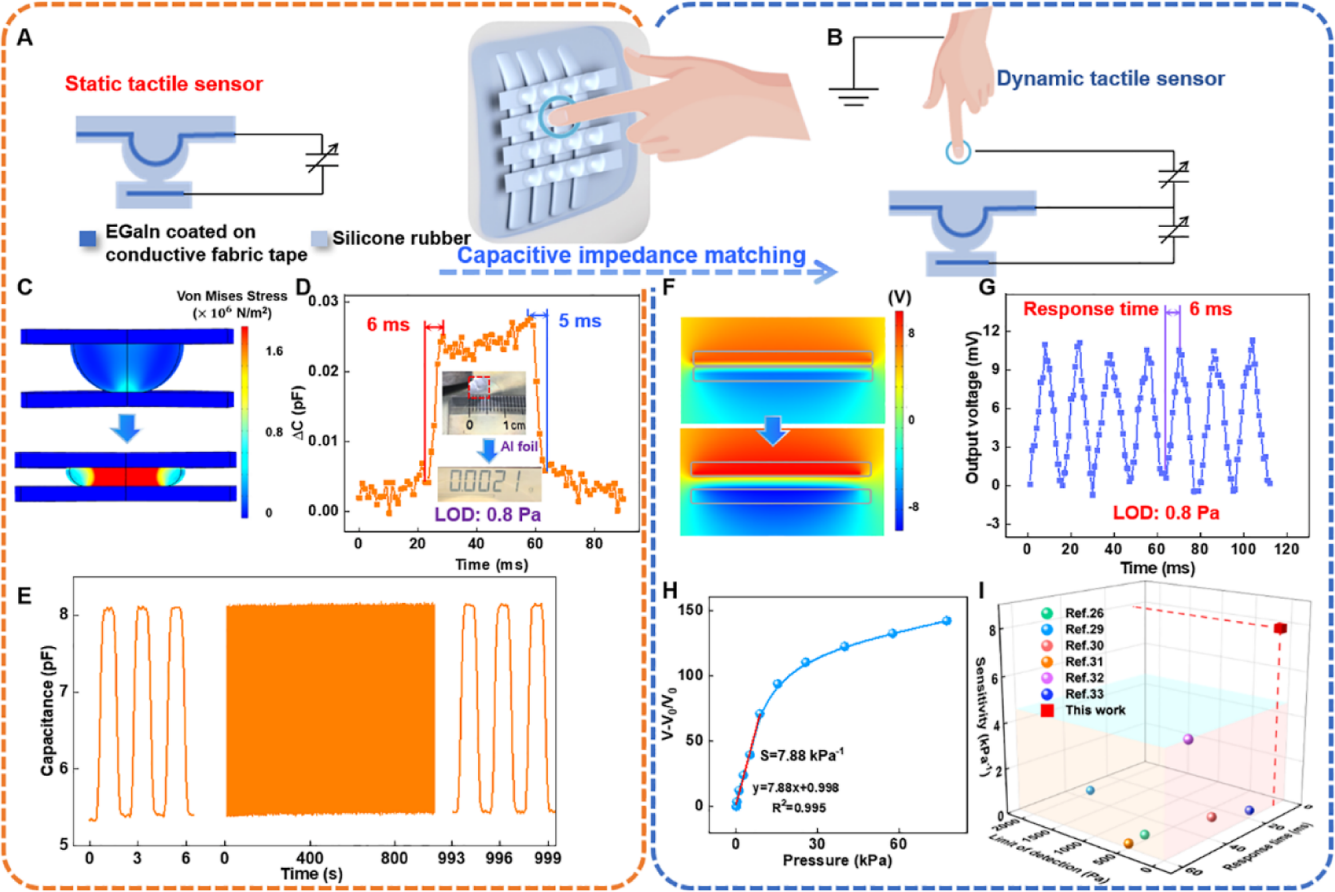
Sensing mechanism and electrical output characteristics of the TCTS array. (A) Proposed TCTS has capabilities to detect static tactile pressure in capacitive sensing mode and (B) dynamic tactile pressure in triboelectric sensing mode. (C) Simulation showing the deformation of the TCTS unit before and after mechanical pressure. (D) Capacitive response and recovery at the static detection limit of 0.8 Pa. (E) Cycle test on capacitance variation under a continuous mechanical pressure of 15.34 kPa. (F) Potential simulation results before and after contact electrification. (G) Voltage response at the dynamic detection limit of 0.8 Pa. (H) Dynamic pressure sensitivity under different pressure loads varying from 0 to 80 kPa. (I) Comparison of this work to other recent research on tactile sensor arrays on three key metrics of sensitivity, response time, and detection limit.^28−33^

The structural design of the dual-mode TCTS array is shown in Figure 2A,B. The TCTS can be implemented separately as a capacitive sensor array for static load distribution sensing as well as a triboelectric sensor for dynamic pressure variation detection. As a capacitive static sensing unit, the top electrode layer is a striped arch electrode coated with EGaIn, and the bottom electrode is a cross-aligned EGaIn planar electrode. Silicone rubber is employed as the dielectric and encapsulation layer. The size of the individual sensor array unit is 7 × 7 mm. The expression for the capacitance of the array unit can be presented as , where *C*_*s*_ is the capacitance of silicone rubber and *C*_*a*_ is the capacitance of the air between two electrodes. Detailed formulas and derivation process of *C*_*s*_ and *C*_*a*_ are presented in Supporting Information Note S1. In the absence of pressure, the separation distance between the two flexible electrodes is at its maximum, and the contact area is at its minimum. An increase in externally applied pressure causes a larger shape change in the microstructure, which leads to a deformation of the embedded electrodes, thus decreasing the separation distance, increasing the area of the contact area, and contributing to a larger capacitance value.

Figures 2C and S3 illustrate the simulation result of the change in deformity about the unit before and after a mechanical pressure of 15.34 kPa is applied. The increase in applied pressure driven by the linear motor results in an increase in the compressed depth of the capacitive sensor unit. Figure 2D depicts the static capacitive sensing unit with a low detection limit of 0.8 Pa and a fast response time of 6 ms. Specifically, the recovery time of the sensor is approximately 5 ms, attributed to the low Young’s modulus and viscoelastic properties of the Ecoflex elastomer and the absence of external forces during the recovery process, collectively enabling a more efficient and rapid restoration of the initial capacitance value. A cycle test on capacitance variation under a continuous mechanical pressure of 15.34 kPa shown in Figure 2E reveals the excellent stability of the sensing unit. Regarding the triboelectric sensor as the mode of dynamic pressure detection, electrical outputs can be detected from two EGaIn electrodes during the process that the finger contacts and separates from the top silicone rubber layer. As depicted in Figure S4, the equivalent circuit diagram of the all-in-one TCTS sensing unit can be represented as the connection of a single-electrode mode TENG and a variable load capacitance. The upper layer of silicone rubber wrapping the arched EGaIn electrode forms a single-electrode TENG, while the upper and lower layers are stacked to acquire a load capacitive. Therefore, the measured electrical output voltage is the divided voltage across the capacitive sensor. A cycle of the electricity generation process for illustrating the working mechanism of the single-electrode mode TENG is shown in Figure S5. Based on the coupling effect of contact electrification and electrostatic induction, an alternating current signal is generated during the contact separation of the finger and the TCTS unit, and the outcome of the simulated potential variation is shown in Figures 2F and S6. To further investigate the working mechanism of the TCTS unit, considering the intrinsic capacitor model and capacitive impedance matching effect of the TENG, various load capacitances were connected in parallel with the TCTS, and electrical output voltages at two ends of different load capacitances were measured, as shown in Figure S7. From previous research, it can be assumed that the inherent impedance of TENG is infinitely large and there is no resistor in the circuit. When the external capacitance value is very small, it reflects the impedance significantly larger than that of the TENG. Therefore, almost all of the open-circuit voltage is loaded on the external capacitance and the TENG works in the quasi-open circuit condition. Since the capacitance variation range is lower than 20 pF (Region I), the output voltage scarcely changes under different pressures of 1.15, 5.22, 15.34, and 57.68 kPa. In this case, the applied pressure becomes the critical factor affecting the output of TCTS. When a tiny pressure of 0.8 Pa is continuously applied, the voltage signal fluctuates between 0 and 11 mV along with the pressure loading and releasing, directly reflecting the dynamic pressure variation process, as represented in Figure 2G. The baseline noise voltage of the triboelectric sensing mode with no applied pressure is characterized in Figure S8. The signal-to-noise ratio (SNR), calculated as the signal power divided by the noise power, quantifies the sensor output quality. The peak amplitude squared ratio readily estimates SNR. Considering the experiment result shown in Figure 2D, the SNR could be calculated as 15.6 dB for the capacitive sensor, confirming robust signal levels exceeding intrinsic noise. Figure 2H illustrates the relationship between pressure and the relative variation of output voltage. The sensitivity of the TCTS unit could be presented as (*V* – *V*_0_)/*V*_0_. A smooth nonlinear relationship between the sensitivity and applied pressure is shown in the whole sensing range (0–80 kPa). However, in the low-pressure range (0–8.78 kPa), a linear relationship is observed due to the significant deformation of the TCTS unit. As a result, a high sensitivity of 7.88 kPa^–1^ was acquired for the linear fitting relation with the *R*^2^ value of 0.995. Meanwhile, for a better display of the pressure dynamic variation, the output voltage profiles under different pressures are illustrated in Figure S9. Also, the relationship between output voltage and applied pressure of different sensing units in the pressure range of 0–80 kPa is depicted in Figure S10, exhibiting repeatability of TCTS. Considering the possible impact of cross-talk on the electrical outputs of sensing units, the output voltages of different surrounding units no 2, 5, 7, and 10 were measured when the particular sensing unit of no. 6 is compressed at the highest pressure of 80 kPa. As a result, there is a slight enhancement in the output voltage with an average accuracy of 2%, which are depicted in Figure S11 and Table S1. Due to the dissimilarity in electronegativity, triboelectrification with different materials produced specific magnitudes in output voltage, as shown in Figure S12. In comparison, metrics including spatial resolution, number of pixels, response time, sensitivity, and limit of detection, as well as neuromorphic computation enabled artificial intelligence applications to recognition and HMI of our work to other recent research on different types of capacitive or triboelectric tactile sensor arrays are summarized in Table S2.^17,28−35^Figure 2I depicts three key device capacities related to response behavior. It is obvious that the TCTS array demonstrates outstanding device performance and broad application prospects in the field of AIoT.^36^ To characterize the reproducibility, three additional TCTS arrays were fabricated using identical preparation methods and tested under the same conditions. As shown in Figure S13, the output voltages for the triboelectric sensing modality exhibit a standard deviation of 4.4% between devices at a pressure of 78 kPa. Similarly, the initial capacitance value had a standard deviation of 3.9% between the sensors. The small variability between different sensors indicates a high level of reproducibility within the fabrication batch. The experimental characterization of the sensor output was conducted under controlled conditions of 25 °C and 40% RH. Figure S14 demonstrates that the sensor output exhibits dependence on both temperature and humidity, with the magnitude of the influence varying between the two parameters. At 50% RH, when increasing the temperature from 0 to 40 °C, the output voltage of the triboelectric sensor exhibited an approximately 44% reduction under the maximum applied dynamic pressure of 78 kPa. Additionally, the initial capacitance value of the static capacitive sensor showed a decrease of around 9%. Under the condition of 20 °C, the triboelectric sensor exhibited a slight decrease in output voltage of approximately 13%, while the initial capacitance value of the static capacitive sensor showed a small increase of around 7%, with the relative humidity increases from 10 to 90%. This result demonstrates that the increase in temperature leads to a certain effect on the output voltage of the triboelectric sensor, while the increase in humidity has a smaller influence.^37,38^ The initial capacitance value of the capacitive sensor also changes with variations in the temperature and humidity but to a negligible degree.

### Static Pressure Visualization Application Enabled with Capacitive Sensor Array

**Figure 3:**
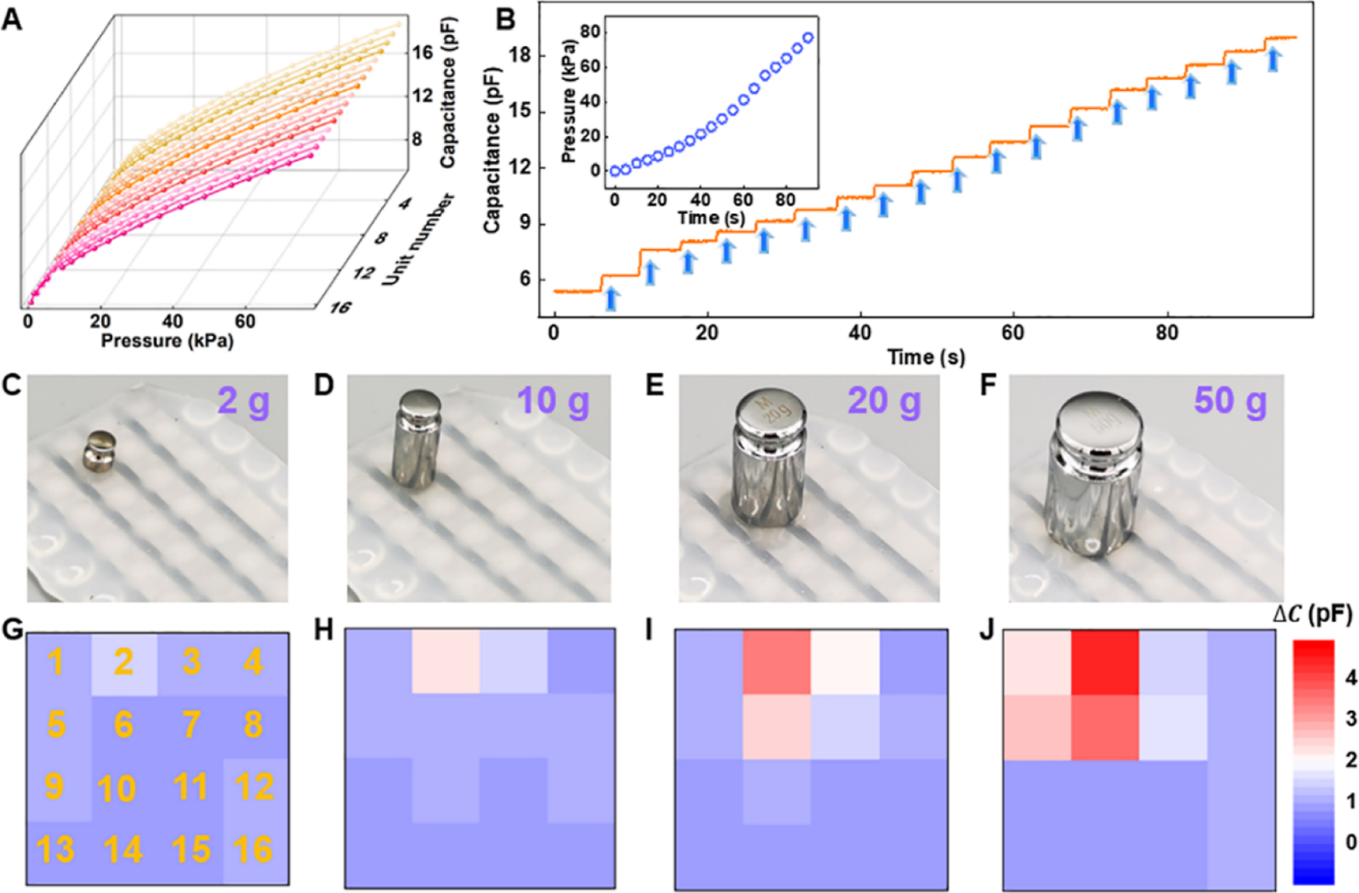
Application in static tactile pressure sensing. (A) Relationship between capacitance and applied pressure for 16 units of TCTS array. (B) Capacitance change and static holding by loading pressures via the linear motor every 5 s. The inset shows the corresponding applied pressures. (C–F) Optical photographs showing the top view of standard weights of 2, 10, 20, and 50 g on the TCTS array. (G–J) Their pressure mapping statistical distribution matrices for 1–16 pixels shown by capacitance variation values.

The relationship between capacitance variation and the applied pressure of all 16 sensing units is illustrated in Figure 3A. The capacitance values increase from ∼5 to ∼19 pF as the pressure strength generally raises to ∼80 kPa, demonstrating excellent device consistency. The capacitive sensor exhibits a high sensitivity of 17% kPa^–1^ from 0 to 1 kPa (Figure S15). Above 1 kPa, the sensitivity gradually decreases as the degree of dielectric deformation reduces. As shown in Figure 3B, the instantaneous change and the long-time static stabilization of the capacitance value can be observed by gradually applying pressures on the sensing unit with a linear motor operating in the same step and maintaining it for 5 s at a time, showing the static pressure detection possibility. The inset shows the detailed applied pressures. Moreover, the flexible capacitive mode pressure sensor array is capable of detecting static pressure signals accurately with high resolution. It can display static visualization of the pressure distribution, reflecting slight stress variability of different areas. As shown in Figure 3C–F, different weights of 2, 10, 20, and 50 g were placed in the top left corner position of the sensor array. The bottom diameters of the 2, 10, 20, and 50 g weights are 0.55 0.9, 1.3, and 1.8 cm, respectively. Since the sensing unit measures 7 mm in diameter and the rows are positioned 2 mm from the edges, a 2 g weight can be entirely located on top of one sensing unit, and a 10 g weight covers one sensing unit and its edges. Moreover, a weight of 20 g extends to the three nearby sensing units, and a weight of 50 g concentrates its pressure on the middle two sensing units and partially dissipates it over the four adjacent sensing units. Figure 3G describes the capacitance variation matrix of the sensor array after situating the 2 g weight, portrayed by a color mapping from blue to red, indicating the magnitude of capacitance change of each pixel for pressure perception. The capacitance variation value of sensing unit no. 2 directly under the 2 and 10 g weights are 1.16 and 2.71 pF, respectively, as represented in Figure 3H. Changes in capacitance at the relevant locations when putting the 20 g weight on the sensor array are 3.74 pF (unit no. 2), 2.13 pF (unit no. 6), and 1.66 pF (unit no. 3) (Figure 3I). The pressure distribution of the 50 g weight located on the sensor array is most obvious, with the corresponding 6 pixels presenting capacitance variations of 4.98 pF (unit no. 2), 3.91 pF (unit no. 6), 2.55 pF (unit no. 5), 2.12 pF (unit no. 1), 1.36 pF (unit no. 7), and 0.90 pF (unit no. 3) (Figure 3J), realizing the application of static pressure mapping pattern visualization.

### Dynamic Pressure Detection and Its Application in Neuromorphic Recognition

**Figure 4:**
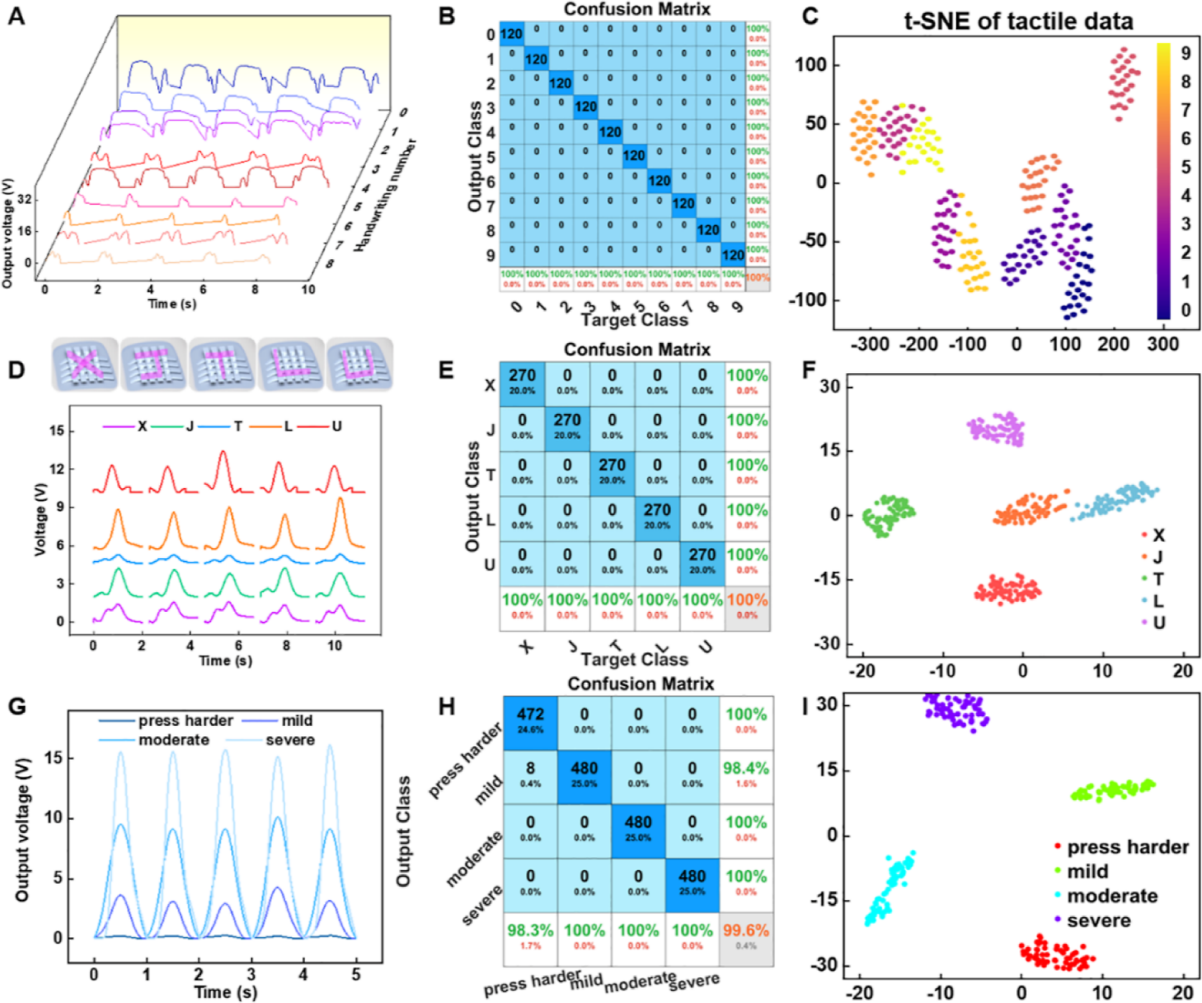
Tactile neuromorphic system for dynamic handwriting and pressure recognition. (A) Output voltage curves and (B) confusion matrix between actual handwriting input and predicted handwriting output signals after the 80th training epoch of handwriting numbers from 0 to 9. (C) Visualization of the somatosensory information using the t-distributed stochastic neighbor embedding (t-SNE) dimensionality reduction method. (D) Output voltage curves, (E) confusion matrix, and (F) t-SNE result for handwriting letters of “X,” “J,” “T,” “L,” and “U” after 55 training epochs. (G) Output voltages curves, (H) confusion matrix, and (I) t-SNE result identifying different pressure strengths, namely, “press harder,” “mild,” “moderate,” and “severe”, after the 75th training epoch.

The development of neuromorphic computation mainly involves artificial neural networks and hardware-based neuromorphic devices, which possess the advantages of high efficiency, ultralow power consumption, integrated storage and computation capabilities, etc. At the biological level, neurons and synapses are essential building blocks for information processing in the brain. The synapse is the connection between the axon terminal and dendrites of another neuron. The synaptic gap allows for the transmission of neurotransmission from the presynaptic neuron to the postsynaptic neuron, thus acting on the corresponding receptors on the cell membrane and generating synaptic plasticity, which is the most important basis for brain learning and memory. To mimic biological synaptic behavior at the device level, changes in ion concentration of artificial synaptic transistors lead to a channel conductance difference and generate an excitatory postsynaptic current (EPSC). Chemical doping leads to a long-term charge retention property, which results in short-term plasticity gradually shifting to long-term plasticity (LTP). Neuromorphic computing based on synaptic devices was employed to verify the application of dynamic handwriting to the TCTS array. The all-in-one synaptic device with the Al/ZnO_*x*_/MXenes/AlO_*x*_-Li/Si/Al structure is proposed in this work to achieve synaptic plasticity by stimulating the presynaptic terminal. To verify the synaptic plasticity of this device, the nonvolatile conductance is recorded when electrical pulses are applied to the presynaptic terminal, regularly. The paired-pulse facilitation (PPF) preliminarily demonstrates the short-term synaptic plasticity, which is the basic function of biological synapses for processing temporal information between neurons. The fitting curves indicate that the PPF stimulated by five different pulse widths (10, 20, 40, 70, and 100 ms) have a wide range of weight adjustment amplitude (Figure S16). The EPSC behavior of the synaptic transistor can be analyzed by applying a single electric pulse (2 V, 300 ms) (Figure S17). After passing the peak value, the initial conductance slowly recovered and stabilized in 7.7 s. The expression of the PPF index depends on the ratio of the first and second peak values (A_2_/A_1_) of the EPSC (Figure S18). The interval (Δ*t*) between pulses is 60 ms, and the amplitude of electric pulses is 2.0 V. The EPSC and IPSC characteristics obtained by positive/negative pulses are the core components to raise and decrease the synaptic weight in the neural network (Figure S19). Significantly, to apply the synaptic plasticity of the electric synapse to neural networks, the LTP/LTD process is normalized to simulate the weight iteration of neuromorphic computing in cross-array. Each row receives the processed input data, and the conductance difference of each adjacent two columns in the cross-array represents one neuron. LTP/LTD is similar to the weight update manual for neuromorphic computing (Figure S20). The rules of the simulated weight matrix are updated according to the periodic trend of LTP/LTD in the synaptic transistor. Weight is the bridge between two neurons that controls the speed of information exchange. The trained neural network with an iterative update array is designed to complete the identification task (Figure S21). Each weight value in the synaptic array matrix depends on the normalized LTP/LTD trend of neural devices. ANN is based on simulated neurons and synapses, which can be modeled on the synaptic transistor device. Therefore, a single-layer-perception (SLP)-based ANN with a back-propagation algorithm using MATLAB software was established, as shown in Figure S22. A concise 10-category of identification is performed, where 2500 data points of the corresponding waveforms for different handwriting numbers are extracted as input to the ANN. Furthermore, an additional bias voltage *V*_0_ is used as an input to act as a constant term. The weight on each synapse is continually updated between the nodes in the ANN to realize the most applicable association between input data sets and predicted output classifications. Moreover, each synapse contains two synaptic transistors, and the difference in conductance between the two devices is defined as the synaptic weight. For dynamic tactile recognition applications, the TCTS array can operate as a flexible handwriting panel to perceive user input signals, including handwriting numbers, letters, and touch strength. The upper and lower four electrodes of the TCTS array are combined to form two input terminals connected to the test system. The user repeatedly wrote down different numbers from 0 to 9 on the TCTS array, and the recorded output voltages were utilized as the data sets. As shown in Figure 4A, although the tactile strength and frequency of one handwriting number cannot be exactly controlled the same at each time, the voltage output curves of the ten handwritten digits from 0 to 9 differ from each other in terms of peak value, peak width, and peak number. Through the synaptic transistor-based ANN neuromorphic algorithm, the confusion matrix shown in Figure 4B reveals a classification accuracy of 100%. Moreover, t-SNE is employed to reduce the dimensionality of complicated data sets, and a two-dimensional coordinate system of handwritten numbers is presented in Figure 4C. The same category of data is aggregated and distinguished from other categories, showing a high level of visualization. Similarly, the electrical output voltage curves of different handwriting letters are shown in Figure 4D, and the top diagrammatic sketch displays the writing track of “X”, “J”, “T”, “L”, and “U”, respectively. As a consequence, upon 450 training samples (62.5%) and 270 test samples (37.5%) of handwriting letters in the data set, a high recognition accuracy is achieved with the value of 100%, as shown in the confusion matrix in Figure 4E. The t-SNE results in Figure 4F show that the clusters of the five handwritten letters are distinguishable on the two-dimensional spatial visualization, and there are no overlapping regions. Furthermore, the ANN-based neuromorphic computing method can be used to recognize different pressure strengths. Figure 4G depicts the output voltage diagrams of four pressing strengths of the finger, namely, “press harder” (0–5 kPa), “mild” (5–20 kPa), “moderate” (20–50 kPa), and “severe” (50–80 kPa). After the 75th training epoch, a high recognition rate of 99.6% is acquired, as shown in Figure 4H. As depicted in Figure 4I, the clusters of the four tactile intensities are far from each other in the 2D spatial distribution, indicating intuitive dimensionality reduction and visualization. As illustrated in the recognition rate curves shown in Figure S23, the recognition accuracies achieve more than 99% after less than 100 iterations, showing ultrahigh efficiency of the neuromorphic network computation and great potential for future sensory storage and computing integrated artificial intelligence. As shown in Figure S24, the TCTS sensor array also demonstrates potential applicability as an electronic skin technology for tactile sensing in robotic hands. The capacitive sensing mode enables the mapping of pressure distributions when objects are statically grasped or gripped. Additionally, the triboelectric sensing mode provides dynamic voltage responses during manipulator interactions involving contact, sliding, and friction. This enables real-time recognition of materials, such as aluminum and copper. The integrated electrode design achieves high functional density with dual capacitive and triboelectric sensing modes. Overall, the TCTS array demonstrates potential suitability as artificial tactile skin to enabling tactile feedback and perception in human-mimetic robotic systems.

### Demonstration of Physiotherapy on Foot Reflex in MR

**Figure 5:**
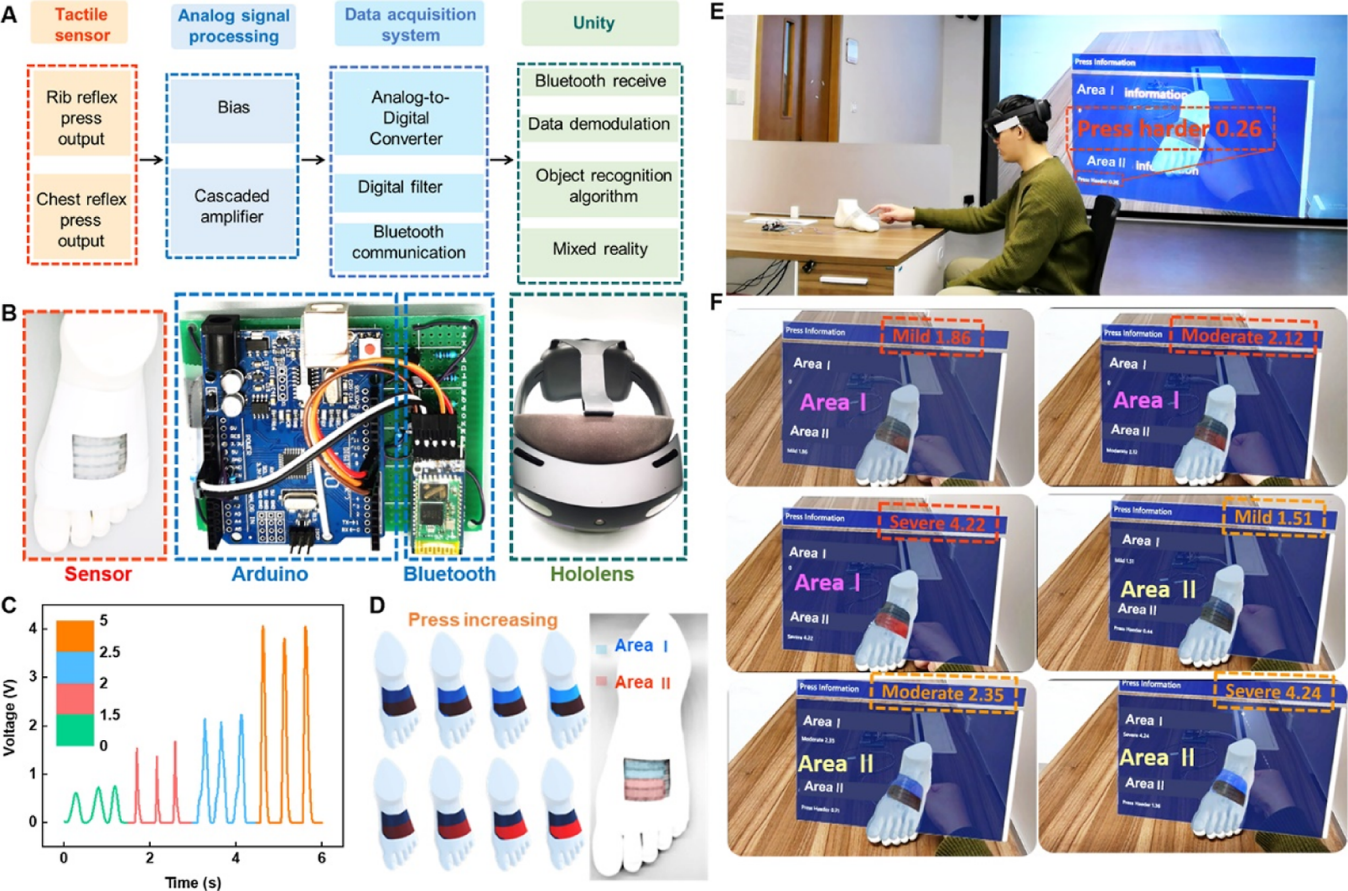
Demonstration of acupressure in MR. (A) System-level block flowchart of the MR-based physiotherapy process, including the acquisition of output voltage signal from TCTS array, analog signal processing, digital converting, and wireless transmission to the custom-developed application in Unity. (B) Optical photographs of the corresponding four modules, namely, the TCTS put on a 3D-printed foot, Arduino for data collecting and analog-to-digital processing, bluetooth for signal transmission, and HoloLens as the terminal display. (C) Output voltage profiles after Arduino processing, presenting four ranges of pressure strengths, especially 0–1.5 V (“press harder”), 1.5–2 V (“mild”), 2–2.5 V (“moderate”), and 2.5–5 V (“severe”). (D) Multichannel visual color mapping of pressing intensity. Area I is indicated by blue, and Area II is indicated by red. (E) Demo of acupressure when placing the TCTS array covering the modeling areas. (F) Different visual colors to distinguish the pressure sensing area and color shades to determine the intensity of pressure.

The emerging MR interface has been recently employed in various applications benefiting from enhanced interaction and an immersive experience, which would be an ideal platform for advanced interpretation, visualization, and communication interfaces. Moreover, the integration of deep learning with the MR interface brings a broad prospect for building an intelligent social network. Therefore, a deep learning-integrated MR interface to realize bidirectional communication was developed, as shown in Figure 5A. The system comprises four major blocks, including TCTS for tactile sensing signals, the printed circuit board (PCB) for signal preprocessing, the IoT module Arduino and bluetooth for data acquisition, and an MR interface in Unity for interaction. With a pretrained machine learning model, models and hands of the user are recognized and generated as output images. Then the images are rendered in the HoloLens interface as outputs. The optical photographs of TCTS put on a 3D printed foot model, analog signal processing PCB, bluetooth communication, and HoloLens display terminal are shown in Figure 5B. Signal-processed output voltage curves at four pressures are illustrated in Figure 5C, showing four ranges of 0–1.5 V (press harder), 1.5–2 V (mild press), 2–2.5 V (moderate press), and 2.5–5 V (severe press). Figure 5D interprets the visual color mapping of the different pressing intensities. The TCTS is placed on the 3D-printed foot model covering different areas for multichannel signal perception. Area I is indicated by blue, and Area II is indicated by red. The colormap deepens as the pressure increases. The application demonstration of visual acupressure is shown in Figure 5E. The main purpose of utilizing mixed-reality technology for this work is to enhance the tactile perception of acupressure points using visual cues. This approach is supported by research on the use of object detection and tracking algorithms and SDKs in mixed-reality HMDs such as Microsoft HoloLens2. Our prototype enables the analysis of real-time data to detect and track the real-life counterpart (i.e., a 3D printed foot model) on a HoloLens 2 headset. Ultimately, it overlays the virtual foot model and geographically aligned holographic texts and images onto the detected objects. As illustrated in Supporting Information Movie S1, the user can simultaneously observe the virtual projection and real space entity of the 3D foot model through the HoloLens equipment and easily realize accurate model tracking. Both the real scenes and the virtual image, including 3D model feet and different area patterns, are presented. Perfect projection of virtual images on the real 3D foot model is realized, and the TCTS array is attached to the model, covering two areas. As presented in Figure 5F, the visual acupressure effects show different shades in red or blue as responses to the user’s operations, based on the pressing strength and the pressing area. From the user interface, an information board was displayed in front of the user, showing the strength of the press for the two areas in digits. As shown in Supporting Information Movie S2, the feedback display position could be adjusted for a better view via finger control. As demonstrated in Supporting Information Movie S3, when the user presses the area gently, the interface will inform the user to press harder. When the pressure strength grows, the color becomes brighter, indicating different pressure areas and pressure intensities of acupressure.

## Conclusions

In summary, a dual-mode 4 × 4 flexible TCTS array with a spatial resolution of 7 mm is designed, which achieves both stable static pressure distribution recognition in capacitive sensing mode and sensitive dynamic pressure detection in triboelectric sensing mode. Neuromorphic computing based on synaptic devices is employed to verify the application of handwriting on the TCTS array. The difference in the conductivity between the two synaptic transistors is utilized as a synaptic weight. Upon 450 training samples (62.5%) and 270 test samples (37.5%) of handwriting letters in the data set collected by the TCTS array, a high recognition accuracy is achieved with the value of 100% within 60 epochs, realizing recognition of complicated tactile input signals. Furthermore, the neuromorphic computation method can be employed to recognize four different pressure strengths, namely, “press harder”, “mild”, “moderate”, and “severe”, acquiring a high recognition rate of 99.6%. Incorporated with TCTS for tactile sensing input, the PCB for signal preprocessing, IoT module Arduino and bluetooth for data acquisition, and a MR interface in Unity for interaction, the visual color mapping of pressing intensities in two different areas can be accomplished. Hence, a promising approach to actualizing the connection between virtual and reality is developed, forming a multimedia interactive system integrating vision and touch, breaking through the constraints of space, time, and reality.

## Experimental Section

### Fabrication of the Triboelectric Capacitive-Coupled Tactile Sensor Array

The TCTS array is composed of two panels: the top panel of silicone rubber (Ecoflex 00-30, Smooth-On)-encapsulated arch-shaped EGaIn (Ga 75.5% and In 24.5%, Sigma-Aldrich) electrodes with a parallel array pattern and the bottom panel of silicone rubber-encapsulated flat EGaIn electrodes. To obtain the down panel layer, first, components A and B of silicone rubber were mixed evenly in a 1:1 ratio and spin-coated on the silicon wafer, leaving it at room temperature for about 5 h until fully cured. Second, a 0.7 cm wide conductive tape (3MCN4190, 3M) was stuck on the cured silicone rubber surface, covered with a hollow four-row 6 × 0.7 size steel sheet as a template, and used the air pump nozzle to spray EGaIn liquid uniformly on the surface at a distance of 15 cm for 5 s. Third, another layer of silicone rubber was placed on it to form the down panel. Finally, the top panel was then prepared by placing another silicone rubber layer with EGaIn stripes in the second step onto the circular array template with a diameter of 7 mm and vacuuming at negative pressure in the vacuum oven for 30 s. More silicone rubber was then added to the surface for the up-panel encapsulation. The up panel and down panel cross stacking to form a 4 × 4 pixels sensor array.

### Fabrication Process of the Synaptic Transistor

First, a heavily doped Si (*n*^+2^) substrate was cleaned by deionized water and dried under a N_2_ flow. Afterward, the processed substrate was further treated by plasma for 15 min to allow for the film surface hydrophilic treatment. Precursor solutions of AlO_*x*_ [dissolving 2 M Al(NO_3_)_2_·*x*H_2_O in 30 mL 2-methoxy ethanol] and AlO_*x*_-Li [mixing 2 M Al(NO_3_)_2_·*x*H_2_O and 0.20 M lithium hydroxide with 30 mL deionized water] were spin-coated on the substrate at 4000 rpm for 20 s and then annealed for 90 min at 300 °C in the air atmosphere. Then, the MXenes solution was diluted to 1 mg/mL and spin-coated at 4000 rpm for 25 s on the surfaces of AlO_*x*_ and AlO_*x*_-Li films. Substrates with solution films were then oxidized at 80 °C for 1 min on a hot plate in air condition. The ZnO_*x*_ precursor [dissolving Zn(NO_3_)_2_·*x*H_2_O into 30 mL of deionized water] was spin-coated at 4000 rpm for 20 s and then annealed for 2 h at 250 °C in an air atmosphere. The 50 nm thick Al source/drain (S/D) electrodes were fabricated by thermal evaporation through the shadow mask.

### Performance Characterization

The capacitance was measured by an impedance analyzer (6500B, Wayne Kerr) with a driving frequency of 15 kHz. For the electrical output measurement of the TCTS, an external contact force was applied by a commercial linear mechanical motor (Winnemotor, WMUC512075-06-X), and the applied force was detected by digital force measurement (Chatillon, DFS II). A programmable electrometer (Keithley model 6514) was used to test the output signal. The triboelectric potential distribution simulation and mechanical deformation were conducted with COMSOL Multiphysics software. The electrical characteristics of the synaptic transistor were measured with a semiconductor device parameter analyzer (Keysight B1500A).

### Synaptic Transistor-Based Neuromorphic Computation

For ANN-based neuromorphic computing, synaptic weights require both positive and negative values. Therefore, the synaptic weight can be expressed as the difference between each conductance value of two synaptic devices
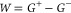
1

During the weight update process, the output vector (y) obtained by the sigmoid activation function was utilized for the above calculation. Δ*W* was then computed using the difference between the output value of the output vector and the label value of the input data set. Then, the positive or negative sign of Δ*W* determines whether the synaptic weight is potentiated or suppressed.

In the case of potentiated synaptic weight, *G*^+^ increases and *G*^–^ simultaneously decreases. Conversely, when in the suppressed synaptic weight phase, *G*^+^ should be decreased while *G*^–^ should be increased. When the conductance of the synaptic device reaches its maximum value (*G*_max_), both *G*^+^ and *G*^–^ are initialized to *G*_min_. The conductance change (Δ*G*) can be calculated according to the following formula
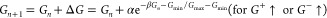
2
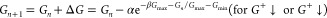
3where *G*_*n*_ and *G*_*n*+1_ represent the current conductance value and the updated value after using the equation, respectively. Furthermore, parameters α and β represent the step size and NL value of conductance change, respectively.

### MR Interface Application in Unity

Unity was used for developing the application, and the overall functionalities were divided into three parts shown in Figure S25: bluetooth communication, input transformation, and computer vision illustration. Data collected from the sensor will be processed into visual effects as MR content. For bluetooth communication, since the system uses bluetooth for communication between the Arduino board and HoloLens, a plugin of Unity, named Arduino bluetooth plugin, was used for the data transportation. All functionalities were implemented in Unity with API given by the plugin. When the two devices are paired, the application records the paired Arduino device on the HoloLens side and starts to listen to the data sent from that device while ignoring the others. Data received will be stored in a queue within the application. For input transformation, data gathered in the queue will be gathered in order and transformed into visual components in a Unity game object. Also, since there are multiple displayed regions, the data are classified into different categories before the transformation. For computer vision illustration, the functionality was implemented based on the MR Toolkit (MRTK) provided by Microsoft, enabling an easier way of developing applications on Microsoft MR devices. The major use of the toolkit is for the calculation of coordinators of projected MR content. Before the projection, images of the environment are captured by cameras and sensors on HoloLens.

## Supporting Information Available

Tracking and matching of virtual and real models (MP4)
Adjusting the feedback display position (MP4)
Color mapping of pressure intensity in mixed reality (MP4)
Detailed formulas and derivation process of dielectric capacitance, optical photograph and fabrication process of the TCTS array, simulation of deformation process under the pressure, equivalent circuit diagram of the TCTS unit, working principle and surface potential simulation of TENG, capacitive impedance matching curve, baseline noise voltage of the triboelectric sensor, output voltage profiles under different pressure loads, relationship between output voltage and applied pressure, cross-talk influence, output voltage curves of different contact materials, reproducibility, influence of temperature and humidity, sensitivity of the capacitive sensor, characteristics of the synaptic transistor, neuromorphic computing process, application in robotic manipulator, and flowchart of application in MR interface (PDF)
