# Supplementary material for: Neuromorphic Computing-Assisted Triboelectric Capacitive-Coupled Tactile Sensor Array for Wireless Mixed Reality Interaction
Source: ACS Nano. 2024 Jun 21;18(26):17041–52. doi: 10.1021/acsnano.4c03554 (PMC11223466; doi:10.1021/acsnano.4c03554)
Supplement: Supplementary file 4 — nn4c03554_si_004.pdf [file nn4c03554_si_004.pdf]

Supporting Information

Neuromorphic-Computing-Assisted Triboelectric-Capacitive-Coupled Tactile Sensor Array for Wireless Mixed Reality Interaction

Xinkai Xie[1, 2, 4, 8 †], Qinan Wang[2, 4, †], Chun Zhao[2,], Qilei Sun[2], Haicheng Gu[1], Junyan Li[2, 4], Xin Tu[4], Baoqing Nie[5], Xuhui Sun[1], Yina Liu[3], Eng Gee Lim[2], Zhen Wen[1, *], and Zhong Lin Wang[6, 7, *]*

1 Institute of Functional Nano and Soft Materials (FUNSOM), Joint International Research Laboratory of Carbon-Based Functional Materials and Devices, Soochow University, Suzhou 215123, P.R. China.

2 Department of Electrical and Electronic Engineering, School of Advanced Technology, Xi'an Jiaotong-Liverpool University, Suzhou 215123, P.R. China.

3 Department of Applied Mathematics, School of Mathematics and Physics, Xi'an Jiaotong-Liverpool University, Suzhou 215123, P.R. China.

4 Department of Electrical and Electronic Engineering, University of Liverpool, Liverpool L693GJ, United Kingdom.

5 School of Electronic and Information Engineering, Soochow University, Suzhou, 215006, P.R. China

6 Beijing Institute of Nanoenergy and Nanosystems, Chinese Academy of Sciences, Beijing 101400, P.R. China.

7 School of Materials Science and Engineering, Georgia Institute of Technology, Atlanta, GA 30332-0245, USA.

8 Joint International Research Laboratory of Information Display and Visualization, School of Electronic Science and Engineering, Southeast University, Nanjing 210096, P.R. China

* Corresponding Authors: chun.zhao@xjtlu.edu.cn (C. Zhao); wenzhen2011@suda.edu.cn (Z. Wen); zhong.wang@mse.gatech.edu (Z. L. Wang)

†These authors contributed equally to this work.

Supporting Information Note S1

In the initial state without applied pressure, the contact points of the upper and lower silicone rubber dielectric layers are defined as the origin of the coordinates and the plane right-angle coordinates are established as shown in Figure S1. The arch shaped part of the sensing unit can be approximated as a hemispherical structure, where r is the radius of the hemisphere. A point m (x,d) on the surface of the upper silicone rubber layer is taken as an example in the initial state. When pressure acts on the sensing unit, the displacement of point m in the vertical direction is defined as y. In this case, the vertical distance between the upper and lower silicone rubber layers at point m can be expressed as:

$$d = r - \sqrt{r^2 - x^2} - y$$

The area of the sensing unit surface in the arch shaped part at the height where the point m (x,d) located can be expressed as

$$dA = \pi x^2 - \pi(x - dx)^2$$

The total capacitance between two electrodes could be equated to the series capacitance of air and silicone rubber, both of which performing as the dielectric media. The formula is presented as:

$$C = \frac{C_s C_a}{C_s + C_a}$$

where C_s is the capacitance of silicone rubber and C_a is the capacitance of the air between two electrodes.

In this case, the specific capacitance expressions of C_s and C_a are presented in the following:

$$C_s = \varepsilon_0 \varepsilon_r \frac{S}{2d_0}$$

$$C_a = \varepsilon_0 \int \frac{\pi x^2 - \pi(x - dx)^2}{r - \sqrt{r^2 - x^2} - y}$$

where ε_0 is the vacuum permittivity, ε_r is the relative permittivity of silicone rubber, S is the effective overlapping area between two electrodes and d_0 is the thickness of silicone rubber dielectric layer.

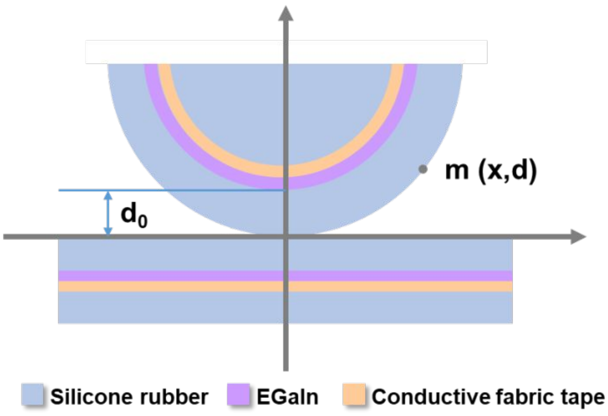

Capacitance model of the sensing array unit

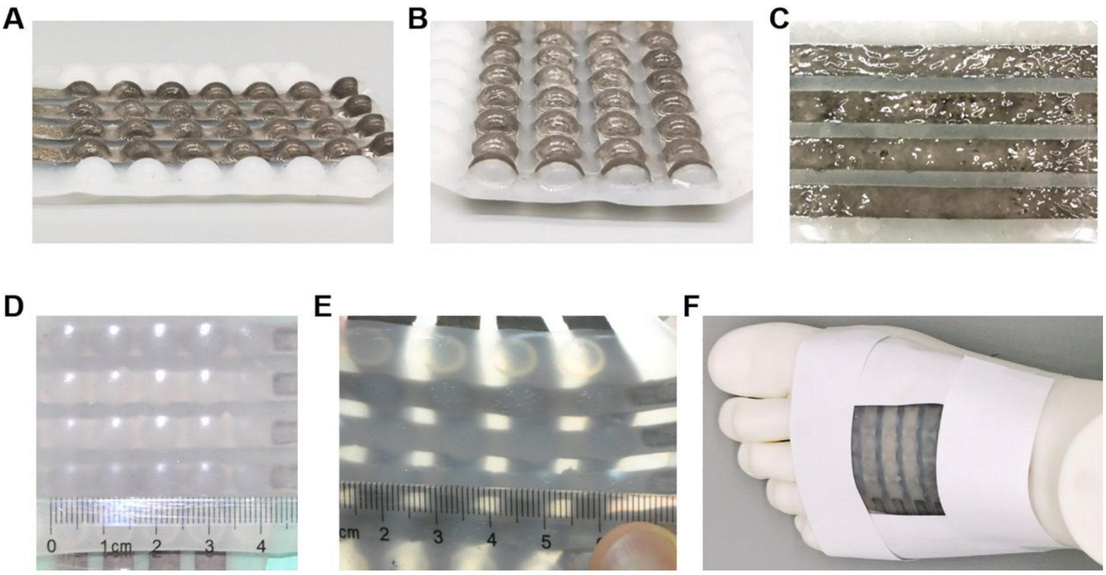

Figure S1. Optical photograph of the TCTS array: (A) Side and (B) front view of the up panel. (C) Top view of the down panel. (D)Top view of the whole device before and (E) after stretching to 140%. (F) TCTS array put on the 3D printed foot model.

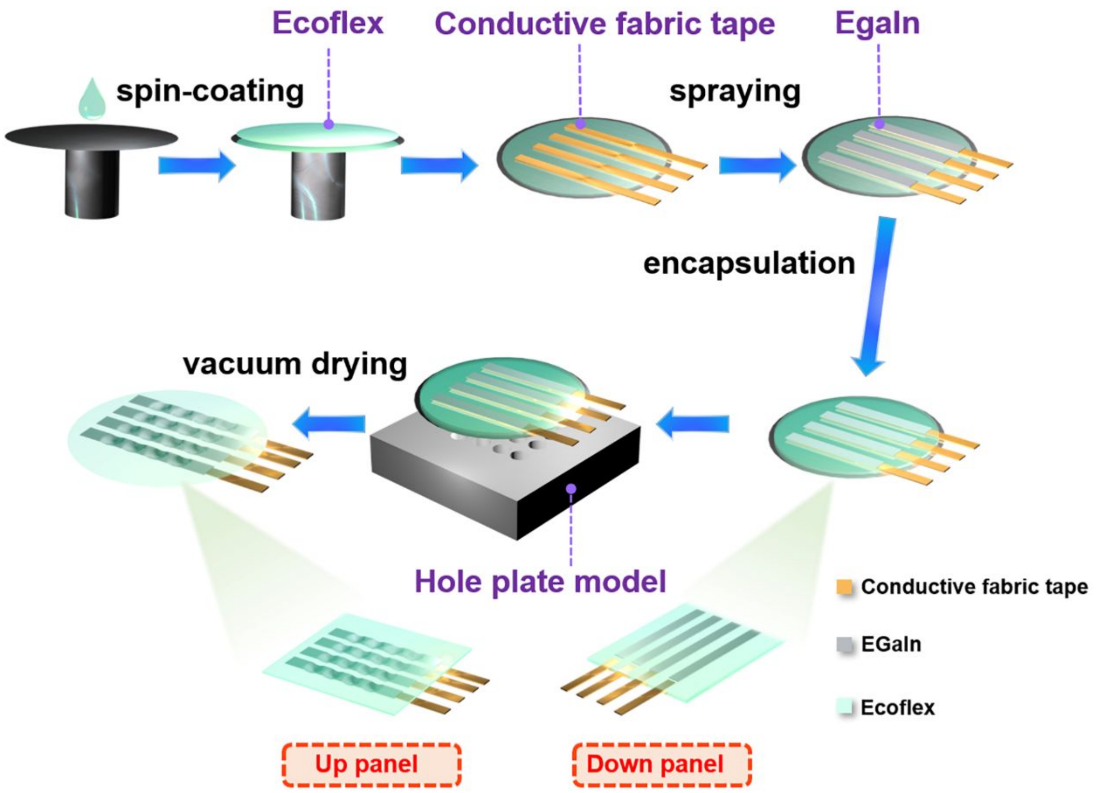

Figure S2. Fabrication process of the TCTS array.

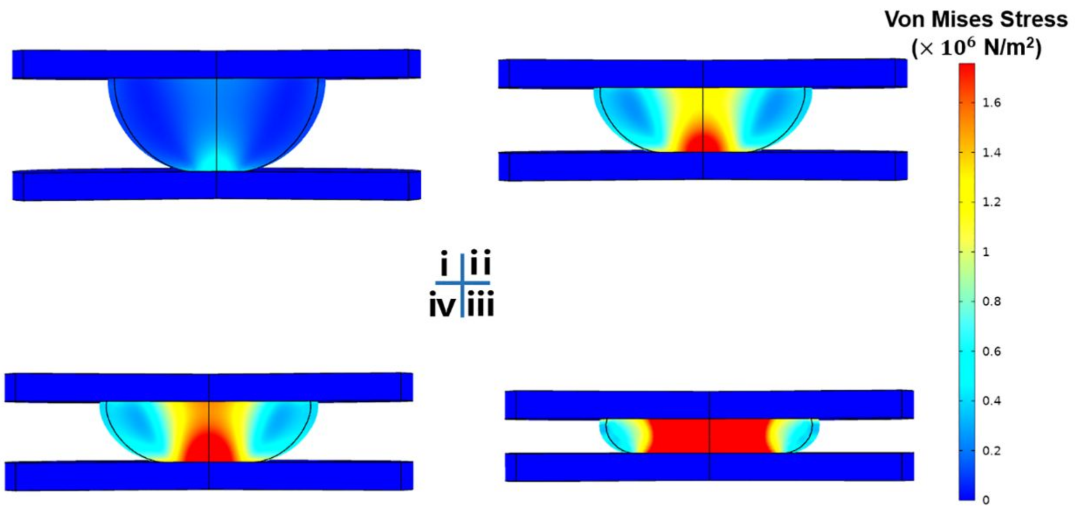

Figure S3. Simulation showing the deformation process of the TCTS unit under mechanical pressure of 15.34 kPa.

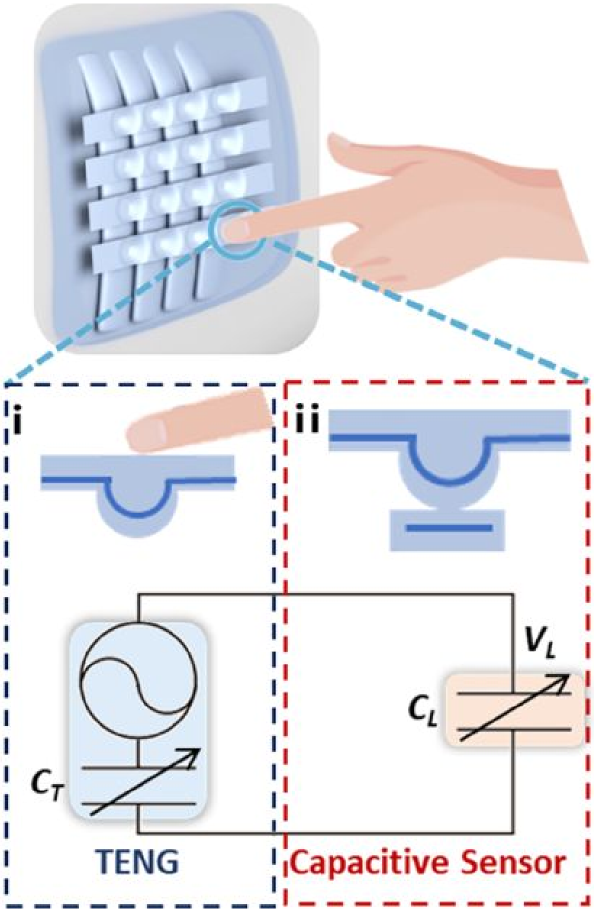

Figure S4. Equivalent circuit diagram of the TCTS unit composed of a load capacitance connected with a single-electrode-mode TENG.

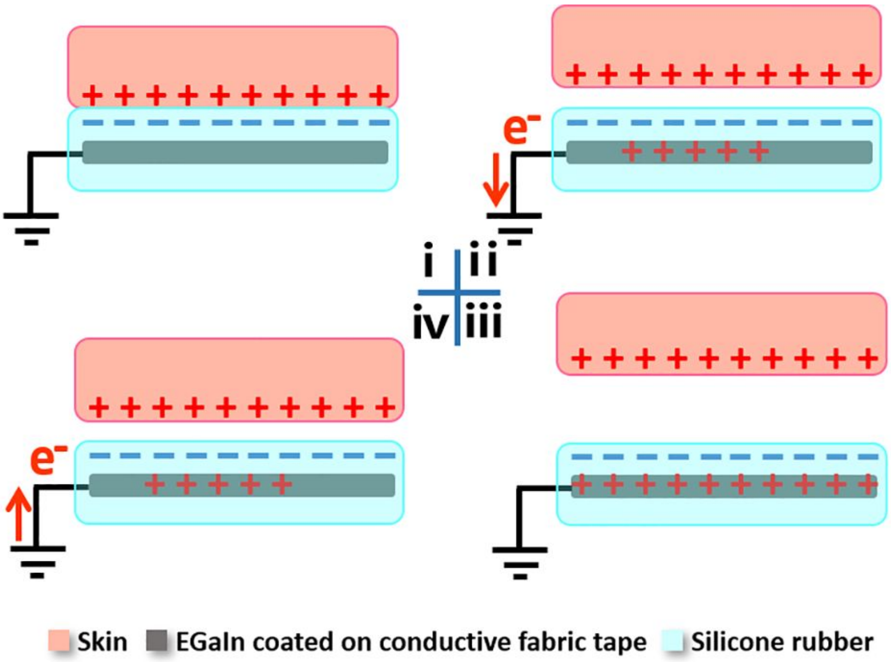

Figure S5. A cycle of electricity generation process for illustrating the working mechanism of the single-electrode mode TENG.

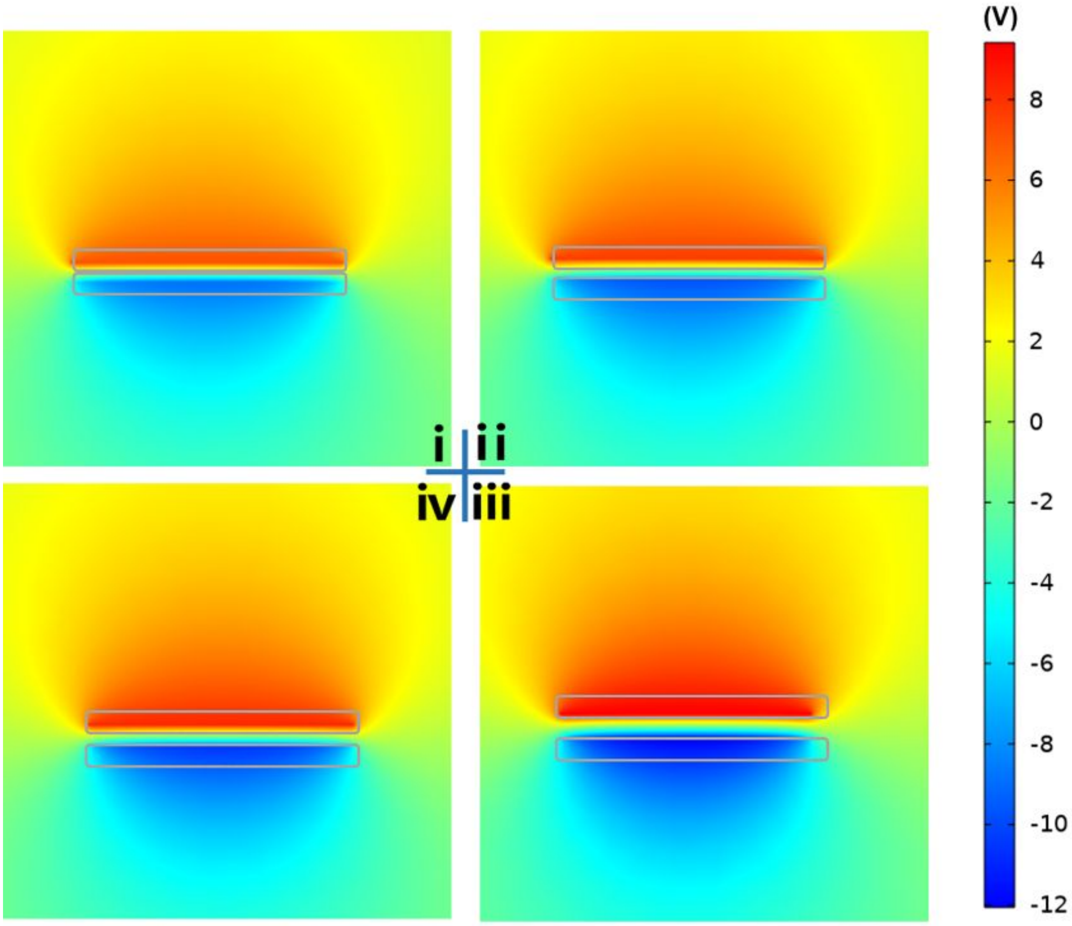

Figure S6. Surface potential simulation during an operation cycle process of the single-electrode mode TENG.

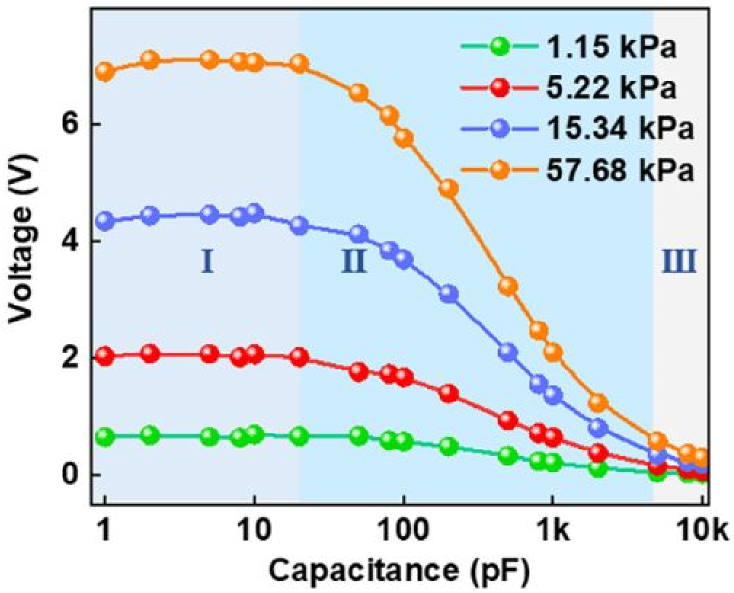

Figure S7. Capacitive matching effect of the single-electrode-mode TENG under various pressures of 1.15 kPa, 5.22 kPa,15.34 kPa, and 57.68 kPa.

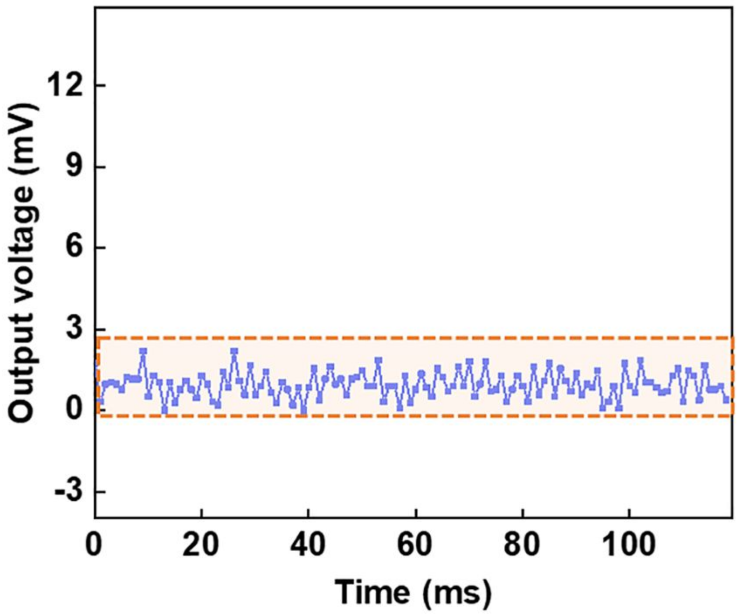

Figure S8. Baseline noise voltage of the triboelectric sensor.

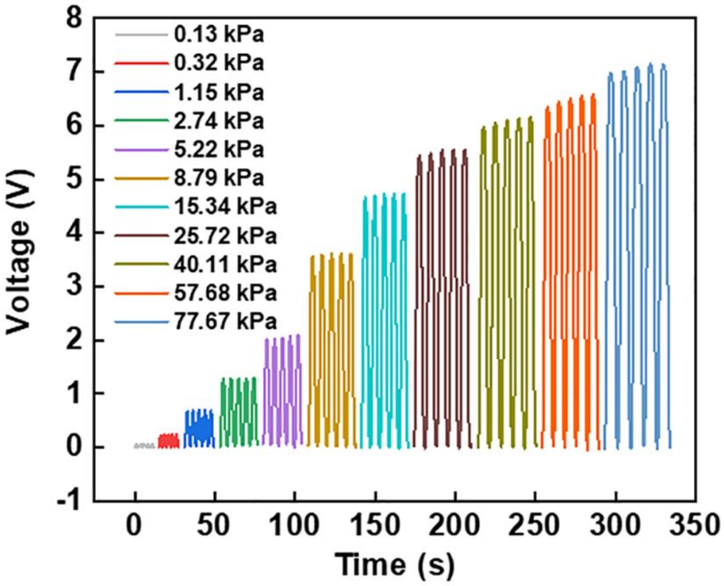

Figure S9. Output voltage profiles under different pressure loads varying from 0-80 kPa.

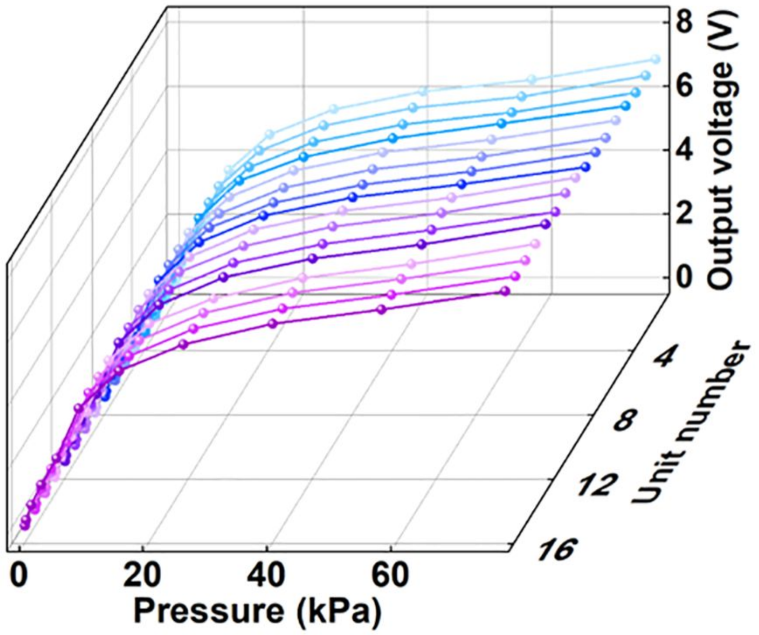

Figure S10. Relationship between output voltage and applied pressure for 16 units of TCTS array.

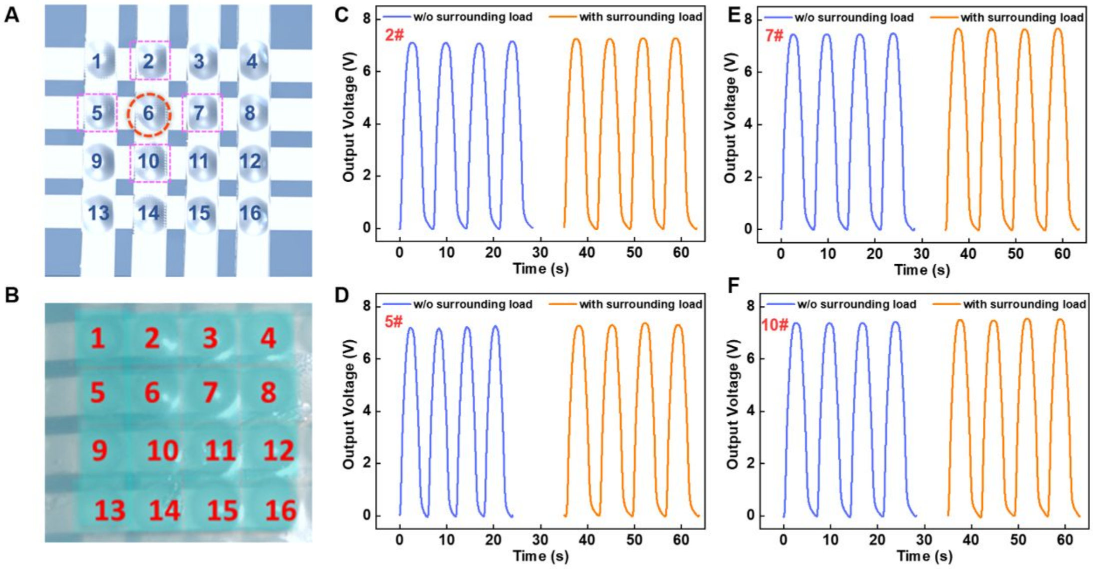

Figure S11. Cross-talk influence. (A) Schematic diagram and (B) optical photographs of the sensing array with labelled units. (C) Comparison in output voltage of surrounding unit No. 2, (D) No. 5, (E) No. 7 and (F)No. 10 before and after unit No. 6 compressed at the highest load of 78 kPa.

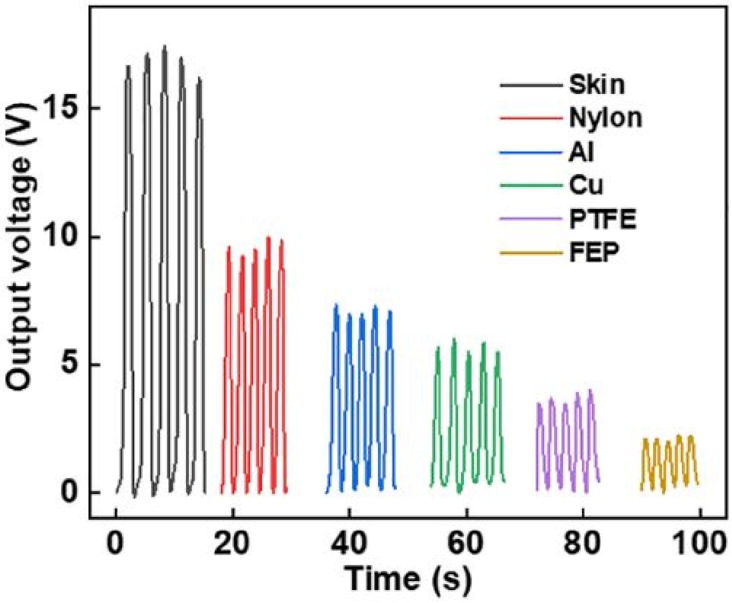

Figure S12. Output voltage curves of different contact materials under the pressure of 15.34 kPa.

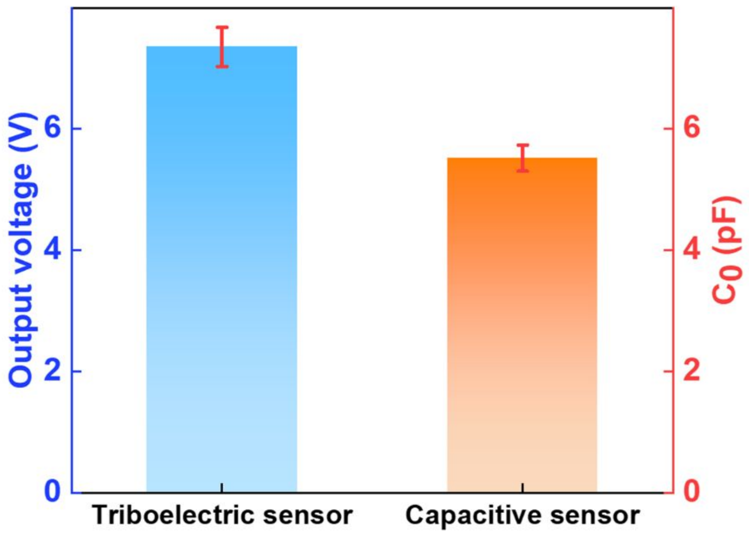

Figure S13. Reproducibility on three additional TCTS arrays.

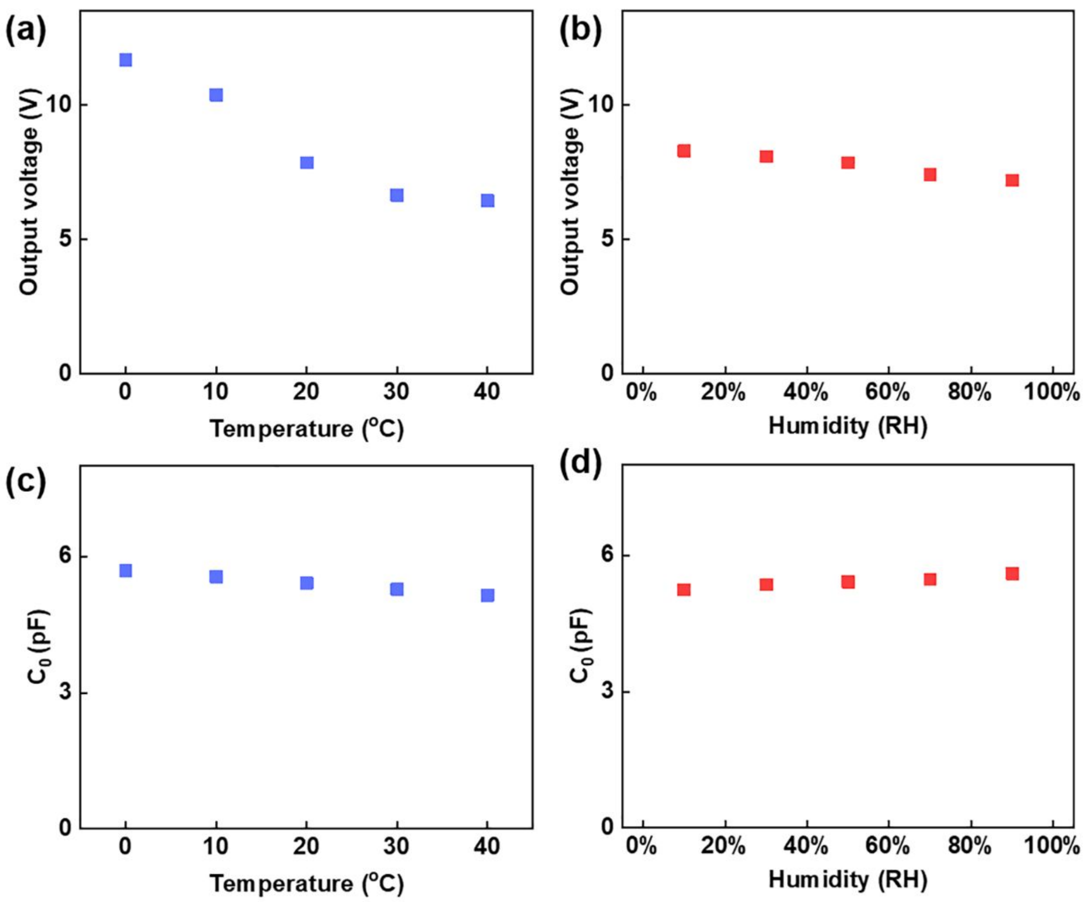

Figure S14. Influence of temperature and humidity in sensor output. Output voltages of the triboelectric sensor at various (a) temperatures (0-40°C) and (b) relative humidities (10%-90%) under the applied pressure of 78 kPa. Initial capacitance values of the capacitive sensor at various (b) temperatures (0-40°C) and (b) relative humidities (10%-90%).

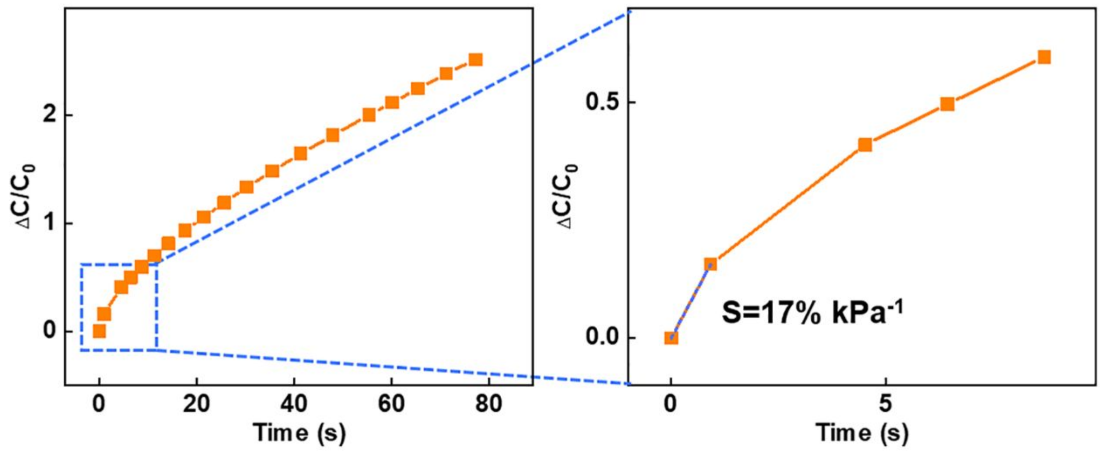

Figure S15. The pressure sensitivity of the capacitive sensor.

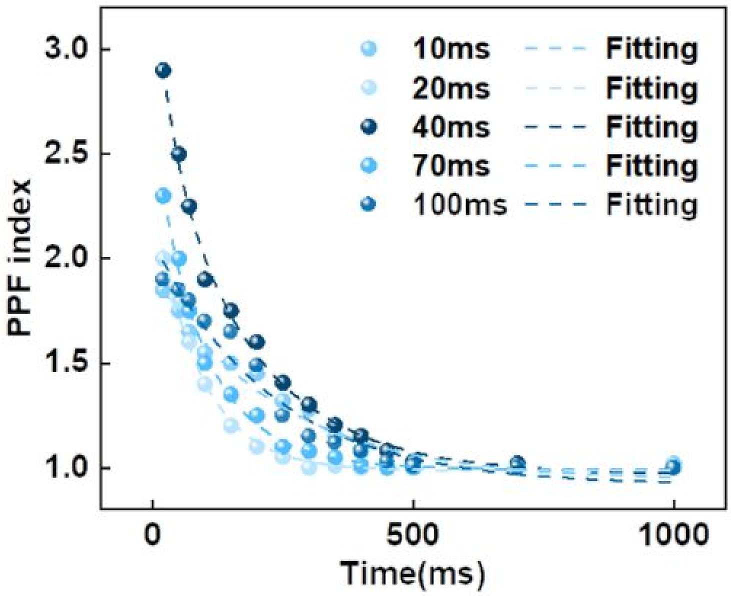

Figure S16. The PPF index indicating short-term plasticity (STP) characteristics of the synaptic transistor.

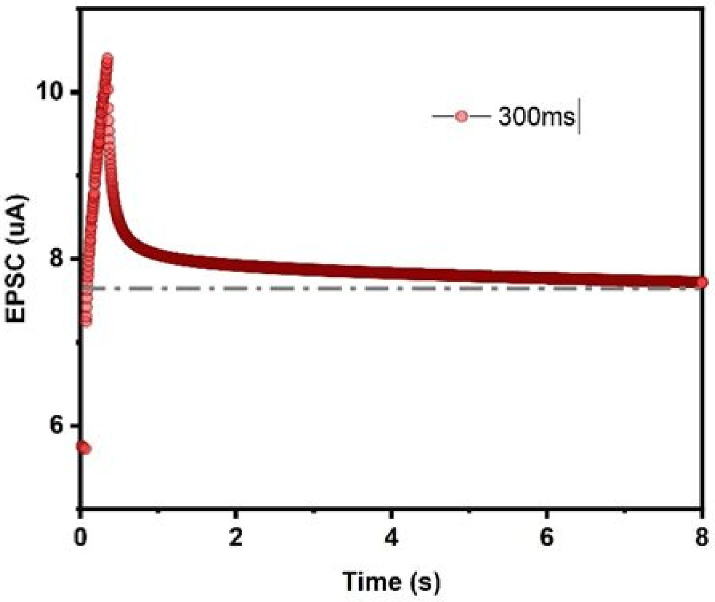

Figure S17. The EPSC behavior of the synaptic transistor.

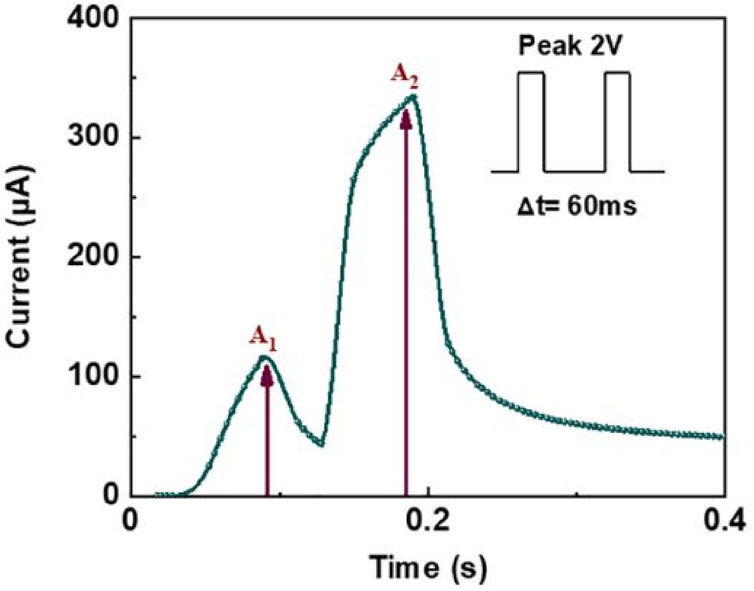

Figure S18. The PPF result of the synaptic transistor.

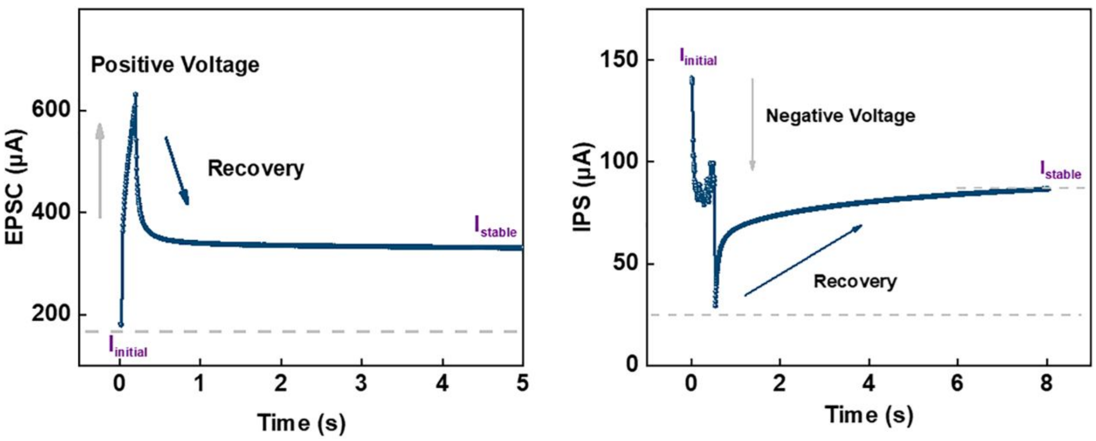

Figure S19. The EPSC and IPSC results of the synaptic transistor during one cycle.

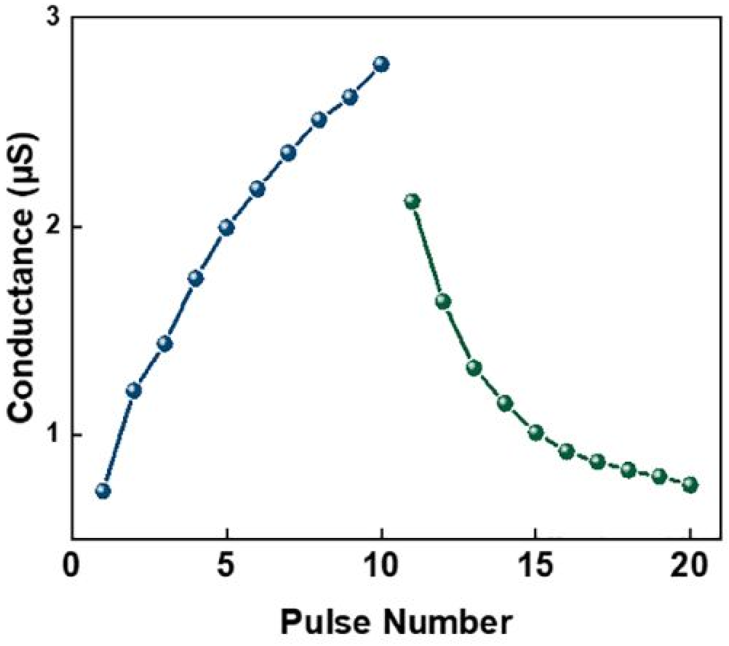

Figure S20. LTP/D characteristic curves as a function of number of pulses.

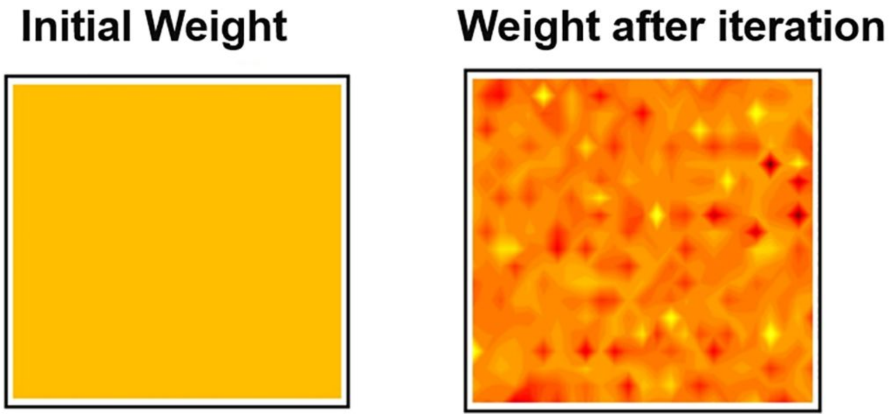

Figure S21. Mapping images of synaptic weights as an increasing of learning iterations.

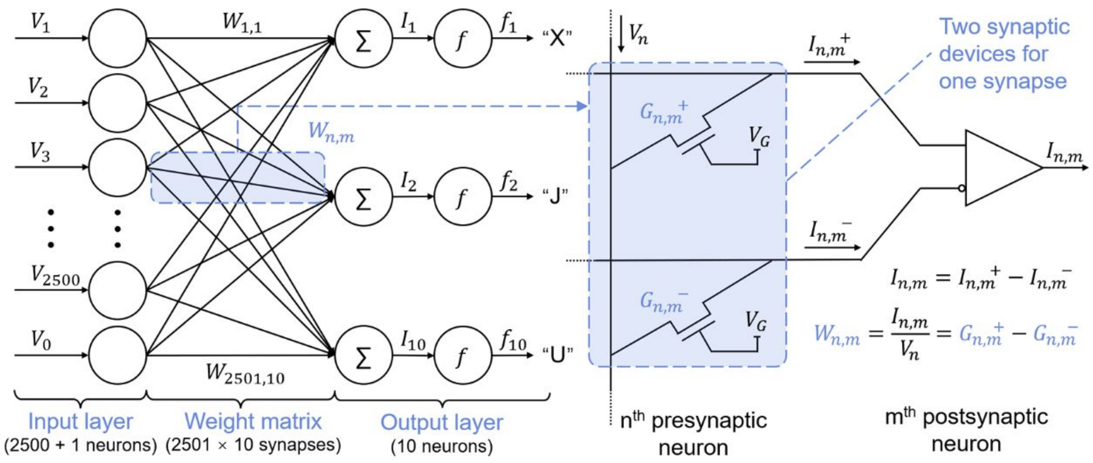

Figure S22. Designed SLP-based ANN with a size of 2501× 10 for the training and recognition simulation. The enlarged view demonstrates the synaptic weight presented by the conductance difference of two equivalent synaptic devices.

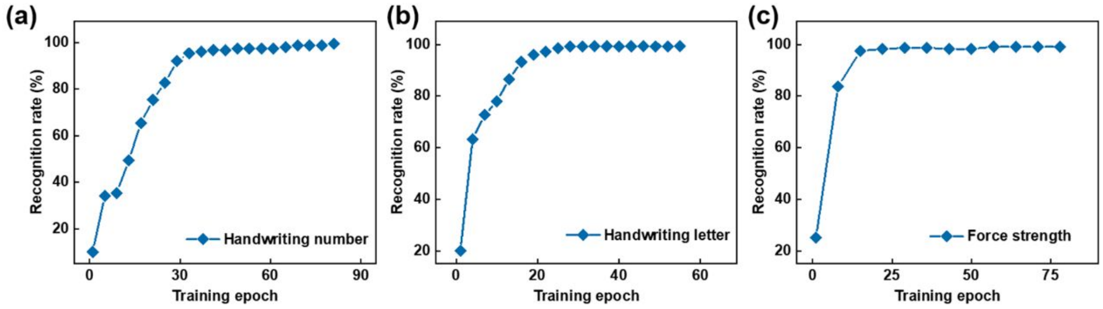

Figure S23. Recognition rate curves of handwriting number, handwriting letter and force strength.

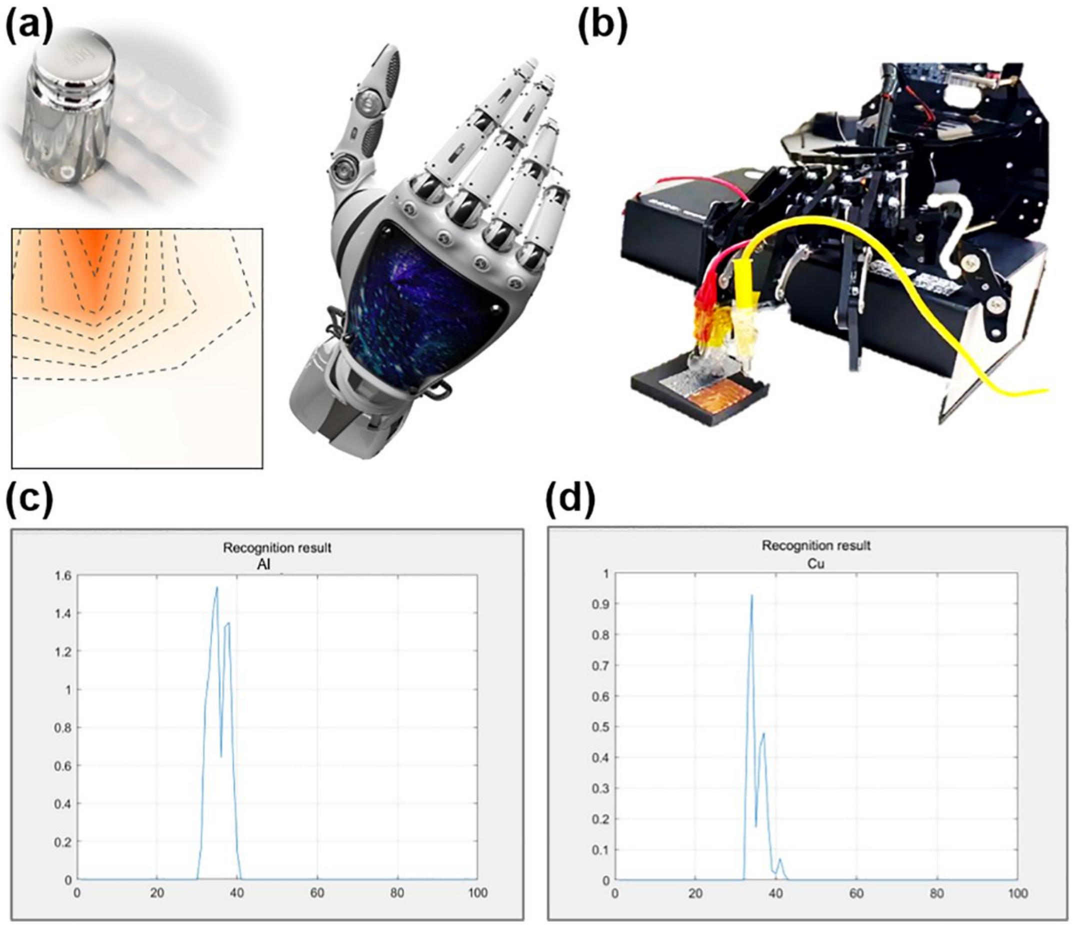

Figure S24. Application in robotic manipulator coupling of static pressure mapping and dynamic pressure-induced material recognition.

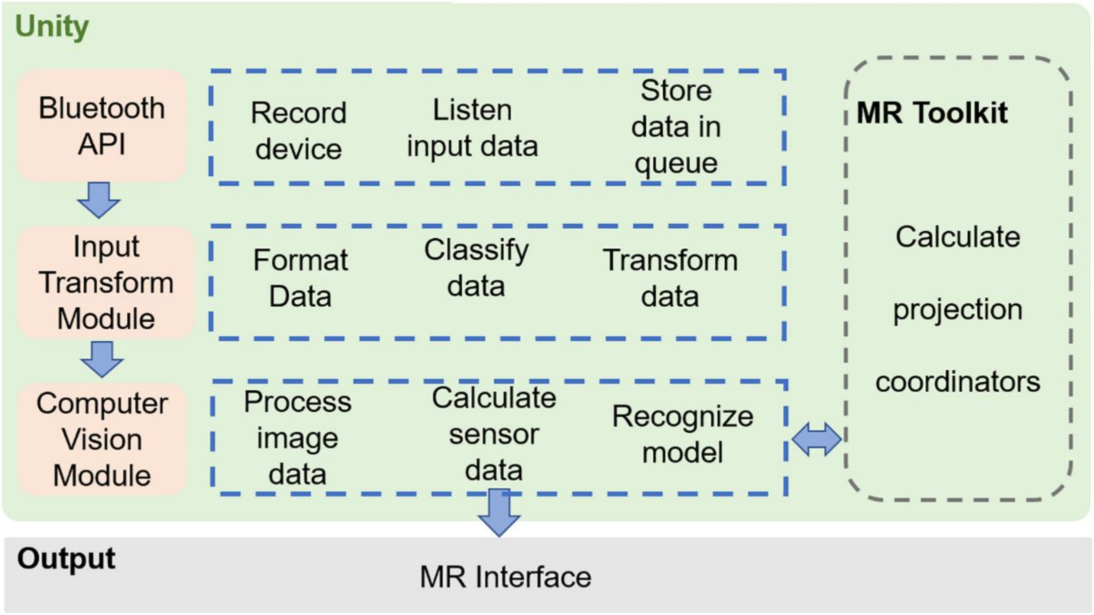

Figure S25. Flow chart of mixed reality interface application in Unity.

Table S1. Statistical results of the electrical output and accuracy analysis affected by cross-talk.

Sensing unit No.	Voltage output w/o surrounding load	Voltage output with surrounding load	Accuracy affected by cross-talk	Average accuracy
2	7.11	7.26	2.1%	2%
5	7.21	7.31	1.4%	
7	7.45	7.65	2.7%	
10	7.38	7.51	1.8%	

Table S2. Comparison on key metrics of recent triboelectric and capacitive tactile sensor arrays.

Ref.	Sensing mechanism	Spatial resolution	# of pixels	Response time	Sensitivity	Limit of detection	Detection range	Application using machine learning algorithm	Application to HMI
28	triboelectric	50 μm*50 μm	100*100	50 ms	6 MPa^{-1}	600 Pa	3 MPa	N/A	N/A
29	triboelectric	15 mm*15 mm	3*3	35 ms	0.08 kPa^{-1} (2-60 kPa)<br>0.008 kPa^{-1} (60-160 kPa)	2 kPa	160 kPa	N/A	N/A
30	capacitive	8 mm*8 mm	4*4	38 ms	0.815 kPa^{-1}(0-1 kPa)	17.5 Pa	0-50 N	N/A	N/A
31	capacitive	90000 μm^2	5*5	60 ms	0.19 kPa^{-1} (<1 kPa)<br>0.1 kPa^{-1} (1–10 kPa)<br>0.04 kPa^{-1} (10–20 kPa)	0.5 kPa	100 kPa	N/A	N/A
32	capacitive	10 mm	3*3	50 ms	4.5 kPa^{-1} (0-1 kPa)<br>2 kPa^{-1}(1-10 kPa)	0.2 Pa	10 kPa	N/A	N/A
33	capacitive	0.8 cm × 0.8 cm	4*4	18 ms	0.24 kPa^{-1} (<70 kPa)<br>1.5 kPa^{-1} (70–150 kPa)<br>0.13 kPa^{-1} (150–330 kPa)	35 Pa	330 kPa	N/A	N/A
34	triboelectric	d=40 mm	7	16 ms	N/A	4 g	N/A	N/A	N/A
17	triboelectric	1.5 cm × 1.5 cm	5*5	N/A	N/A	N/A	N/A	Object identification with accuracy rate of 98.9%	N/A
35	hybrid	10 mm*8 mm	8	N/A	N/A	N/A	N/A	Gestures recognition with accuracy rate of 92.6%	N/A
This work	hybrid	d=7 mm	4*4	6 ms	7.88 kPa^{-1}(0-8.78 kPa)	0.8 Pa	80 kPa	Handwriting recognition with accuracy rate of 100%	MR interfaces
